# Cost-Effectiveness of In-Bed Cycling and Routine Physiotherapy for Patients Receiving Mechanical Ventilation

**DOI:** 10.1001/jamanetworkopen.2025.29399

**Published:** 2025-09-08

**Authors:** Jean-Eric Tarride, Gord Blackhouse, Bram Rochwerg, Alison E. Fox-Robichaud, Ian M. Ball, Karen E. A. Burns, Andrew J. E. Seely, John Muscedere, Susan Berney, Amy M. Pastva, Frédérick D’Aragon, Patrick M. Archambault, Jennifer L. Tsang, Avelino C. Verceles, Karim Serri, Brenda K. Reeve, Shane W. English, Francois Lamontagne, Tim Karachi, Erick H. Duan, Geoff Strong, Laurel Kelly, Julie C. Reid, Jill C. Rudkowski, Heather K. O’Grady, Margaret S. Herridge, Lehana Thabane, Diane Heels-Ansdell, Deborah J. Cook, Michelle E. Kho

**Affiliations:** 1 Department of Health Research Methods, Evidence, and Impact, McMaster University, Hamilton, Ontario, Canada; 2 Centre for Health Economics and Policy Analysis, McMaster University, Hamilton, Ontario, Canada; 3 Department of Medicine, Faculty of Health Sciences, McMaster University, Hamilton, Ontario, Canada; 4 Department of Critical Care, Hamilton Health Sciences, Hamilton, Ontario, Canada; 5 Division of Critical Care Medicine and Department of Epidemiology and Biostatistics, Western University, London, Ontario, Canada; 6 Interdepartmental Division of Critical Care, University of Toronto, Toronto, Ontario, Canada; 7 Applied Health Research Centre, Li Ka Shing Knowledge Institute, Unity Health Toronto–St Michael’s Hospital, Toronto, Ontario, Canada; 8 Thoracic Surgery, University of Ottawa, Ottawa, Ontario, Canada; 9 Critical Care Medicine, Ottawa Hospital Research Institute, Ottawa, Ontario, Canada; 10 Department of Critical Care Medicine, Queen’s University, Kingston, Ontario, Canada; 11 Physiotherapy Department, Division of Allied Health, Austin Health, Heidelberg, Australia; 12 Department of Physiotherapy, The University of Melbourne, Parkville, Victoria, Australia; 13 Physical Therapy Division, Department of Orthopedic Surgery, Duke University School of Medicine, Durham, North Carolina; 14 Department of Anesthesiology, Faculty of Medicine and Health Sciences, Université de Sherbrooke, Sherbrooke, Quebec, Canada; 15 Centre de recherche du Centre hospitalier universitaire de Sherbrooke, Sherbrooke, Quebec, Canada; 16 Centre de recherche intégrée pour un système apprenant en santé et services sociaux, Centre intégré de santé et services sociaux de Chaudière-Appalaches, Lévis, Quebec, Canada; 17 Department of Family Medicine and Emergency Medicine, Université Laval, Québec, Quebec, Canada; 18 Niagara Health Knowledge Institute, Niagara Health, St Catharines, Ontario, Canada; 19 Division of Pulmonary, Critical Care and Sleep Medicine, Department of Medicine, University of Maryland, Baltimore; 20 Critical Care Division, Department of Medicine, Centre de Recherche de l’Hôpital du Sacré-Cœur de Montréal, Hôpital Sacré-Coeur de Montréal, Faculté de médecine, Université de Montréal, Montreal, Quebec, Canada; 21 Department of Medicine, Brantford General Hospital, Brantford, Ontario, Canada; 22 Department of Medicine (Critical Care), University of Ottawa, Ottawa, Ontario, Canada; 23 Clinical Epidemiology Program, Ottawa Hospital Research Institute, Ottawa, Ontario, Canada; 24 Département de médecine, Faculté de médecine et des sciences de la santé, Université de Sherbrooke, Sherbrooke, Quebec, Canada; 25 School of Rehabilitation Science, Faculty of Health Sciences, McMaster University, Hamilton, Ontario, Canada; 26 Physiotherapy Department, St Joseph’s Healthcare Hamilton, Hamilton, Ontario, Canada; 27 Interdepartmental Division of Critical Care Medicine, Department of Medicine, Toronto General Research Institute, Institute of Medical Science, University Health Network, University of Toronto, Toronto, Ontario, Canada; 28 Research Institute of St Joe’s Hamilton, Hamilton, Ontario, Canada

## Abstract

**Importance:**

The cost-effectiveness of adding early in-bed cycling to usual physiotherapy among adults receiving mechanical ventilation in the intensive care unit (ICU) compared with usual physiotherapy alone is unknown.

**Objective:**

To evaluate the cost-effectiveness of in-bed cycling plus usual physiotherapy compared with usual therapy alone in the Critical Care Cycling to Improve Lower Extremity Strength (CYCLE) randomized clinical trial.

**Design, Setting, and Participants:**

This trial-based economic evaluation with a 90-day time horizon compared early cycling plus usual physiotherapy vs usual physiotherapy alone from a societal perspective. Adult ICU patients (aged ≥18 years) receiving mechanical ventilation were recruited from 16 ICUs in Canada, the US, and Australia. Enrollment occurred from November 1, 2016, to May 30, 2023, with the last follow-up on August 3, 2023.

**Interventions:**

Intervention group participants were offered 30 minutes per day of cycling in addition to usual physiotherapy on weekdays, starting within the first 4 days of mechanical ventilation. Cycling continued until the patient could march on the spot for 2 consecutive days, ICU discharge, or for 28 days, whichever occurred first. Usual care participants were offered individualized physiotherapy according to local practices and patient alertness.

**Main Outcomes and Measures:**

Differences in costs (in 2024 Canadian dollars [CA$]) and quality-adjusted life-years (QALYs) between the groups were calculated. In the absence of dominance (ie, 1 strategy is associated with higher costs and fewer QALYs), the results were reported in terms of incremental cost per QALY gained.

**Results:**

The CYCLE trial recruited 360 patients (mean [SD] age, 61.5 [15.6] years; 205 male [56.9%]). The estimated per-patient cost associated with providing early in-bed cycling (CA$321) represented 0.5% of the index hospitalization costs (CA$66 554). The per-patient differences in 90-day costs (CA$5841; 95% CI, −CA$7666 to CA$18 797) and QALYs (−0.0009; 95% CI, −0.0185 to 0.0182) between cycling plus usual physiotherapy vs usual physiotherapy alone were not statistically different from 0. The probability of cycling plus usual physiotherapy to be cost-effective was 0.19 at a willingness-to-pay threshold of $50 000 per QALY gained.

**Conclusions and Relevance:**

In this trial-based economic evaluation, the differences in costs and QALYs between adding early in-bed cycling to usual physiotherapy and usual physiotherapy alone for adults receiving mechanical ventilation were not significantly different from 0. These results highlight the need for additional cost-effectiveness studies considering the full body of evidence regarding in-bed cycling for critically ill patients.

## Key Points

**Question:**

What is the cost-effectiveness of in-bed cycling plus usual physiotherapy compared with usual physiotherapy alone among adults receiving invasive mechanical ventilation?

**Findings:**

This economic evaluation including 360 randomized clinical trial participants found that the per-patient cost of acquiring and delivering early in-bed cycling accounted for 0.5% of the index hospitalization costs. There were no statistically significant differences in the per-patient 90-day costs and quality-adjusted life-years between the 2 groups.

**Meaning:**

These findings highlight the need for future economic studies based on the totality of the randomized clinical trial evidence of in-bed cycling for critically ill patients.

## Teaser

This economic evaluation assesses the cost-effectiveness of early in-bed cycling plus usual physiotherapy compared with usual physiotherapy alone among adult patients receiving mechanical ventilation.

## Introduction

More than 20 million individuals worldwide are admitted annually to an intensive care unit (ICU).^1^ Although ICU mortality rates have decreased over time,^2,3^ many survivors of critical illness experience durable physical impairments following hospital discharge.^4,5^ Muscle atrophy, especially in the legs,^6^ occurs rapidly in critically ill patients, accounting for 2% muscle loss per day.^7^ Intensive care unit–based rehabilitation may address these impairments,^8,9^ but invasive mechanical ventilation and sedation are barriers to its implementation.^10^ Among ICU physical rehabilitation exercises, cycle ergometry is particularly attractive, as it targets the legs and can be offered in bed, even while patients receive mechanical ventilation and sedation. A recent meta-analysis of 33 randomized clinical trials (RCTs) comparing cycling to no cycling among critically ill adult patients (aged ≥18 years) admitted to the ICU for more than 24 hours found that cycling may improve physical function and decrease ICU and hospital length of stay (LOS).^11^

Although substantial clinical data exist supporting ICU-based rehabilitation,^8,9^ no studies to our knowledge have conducted a trial-based economic evaluation. To address this gap and provide valuable insights for patients, families, clinicians, and decision makers, we conducted a preplanned economic evaluation alongside the recently published Critical Care Cycling to Improve Lower Extremity Strength (CYCLE) multinational RCT of in-bed cycle ergometry among adult ICU patients receiving mechanical ventilation^12,13^ to evaluate the cost-effectiveness of in-bed cycling plus usual physiotherapy compared with usual physiotherapy alone.

## Methods

This economic evaluation was conducted in accordance with trial-based cost-effectiveness guidelines^14^ and followed the Consolidated Health Economic Evaluation Reporting Standards (CHEERS) 2022 reporting guideline.^15^ CYCLE was approved by the research ethics boards of all participating centers and Clinical Trials Ontario. All participants or their substitute decision makers provided written informed consent. Analyses were performed from a societal perspective^16^ to capture patient and caregiver productivity losses following hospital discharge.

### Setting and Participants

The CYCLE RCT study protocol and clinical results have been previously published.^12,13^ Briefly, adult ICU patients receiving invasive mechanical ventilation were randomized between November 1, 2016, and May 3, 2023, to cycling in addition to usual physiotherapy or usual physiotherapy alone in medical-surgical ICUs in Canada, the US, and Australia.^13^ The last patient follow-up was August 3, 2023.^13^ Study coordinators contacted patients alive at the designated follow-up window to collect self-reported economic and health-related quality-of-life data. Based on LOS data from the CYCLE pilot RCT (median [IQR], 27.0 [13.5-47.0] days for cycling and 25.0 [19.0-45.0] days for routine care),^17^ a time horizon of 90 days after randomization was chosen to conduct the follow-up assessment. The follow-up window was from 80 to 120 days after randomization (hereafter referred to as 90-day follow-up). Although they contributed to the index hospitalization data, we did not collect follow-up data in the vanguard phase of the trial (46 patients enrolled before March 31, 2018) due to insufficient funding.^13^

### Interventions

In the CYCLE RCT, patients randomized to the intervention group were offered 30 minutes of cycling in addition to usual physiotherapy per day on weekdays (ie, 5 days per week), starting within the first 4 days of mechanical ventilation. Cycling continued until the patient could march on the spot for 2 consecutive days, at ICU discharge, or for a maximum of 28 days, whichever occurred first.^12,13^ Patients in the usual physiotherapy group were offered individualized physiotherapy according to local practices and the patient’s alertness^12,13^ and consisted of movements to maintain or improve limb range of motion and strength, in- and out-of-bed mobility, ambulation, and assistance optimizing airway clearance and respiratory function.^18^

### Cost Measures

All costs are presented in 2024 Canadian dollars (CA$) (currency exchange rate of CA$1 = US $0.73). We used the Statistics Canada consumer price index for health and personal care^19^ to inflate cost data to 2024 values, when necessary. The cost of the in-bed cycle ergometer was based on an equipment cost of CA$25 000 and a 5-year amortization.^13^ We surveyed sites in November 2024 to determine the anticipated bike usage per year following CYCLE trial results publication. To estimate the intervention delivery costs, we used trial data to determine the number of physiotherapists involved in providing each cycling or usual physiotherapy session and the total duration of cycling, physiotherapy sessions, and in-bed bike setup and takedown time (as applicable) for each patient. We multiplied the time per patient by the average hourly wage of a staff physiotherapist in Ontario (CA$57.03 per hour,^20^ including 30% benefits) to estimate the intervention delivery costs.

The costs associated with the index hospitalization were based on the number of days in the ICU and on a hospital ward from randomization to hospital discharge. Actual daily patient-level hospital costing data available for 63 study participants recruited at 3 hospitals in Hamilton (Ontario, Canada) were used to derive a daily cost associated with an ICU (CA$3426) or a hospital ward (CA$1120) day. eTable 1 in Supplement 1 presents the characteristics of these 63 patients compared with the other study patients. Since the hospital costing data did not include physician costs, we added these to the hospital stay. Daily fees associated with the service rendered by the intensivist who delivered care for ICU patients (including ventilator support) and those delivered by the most responsible physician following transfer from the ICU to the hospital ward were based on the Ontario Schedule of Benefits^21^ (eTable 2 in Supplement 1). As 10 of 16 ICUs were from Ontario, the most populated Canadian province, we valued health care resource use with Ontario unit costs.

Research coordinators contacted patients alive at their 90-day follow-up after randomization to document health care resource use and patient or caregiver productivity losses since hospital discharge. Specifically, patients were asked whether they had any health care resource use, including hospital readmission (number of admissions and associated LOS), emergency department visits (number of visits), physician specialist or other health care professional (type and number of visits), and/or general practitioner (number of visits) visits. When a patient answered no to the health care use questions, resource use and costs after hospital discharge were assumed to be 0. Similarly, patients who died during the index hospitalization were assigned 0 follow-up costs. Patients who died after hospital discharge were assigned a cost proportional to the time of death after hospital discharge based on 90-day survivor cost data. Patients were also asked whether they spent time (measured in days) in retirement homes, nursing homes, chronic care, long-term care, rehabilitation, or other facilities since hospital discharge.

To estimate productivity costs, we asked patients employed before their index hospitalization whether and when they had returned to work during their 90-day follow-up. Patients reported whether they had received assistance from others to help them with their daily activities, the number of weeks and hours per week of assistance required, and whether caregivers had to take time off from work. Absence from work for patients or caregivers was calculated by multiplying the number of days away from work by the Statistics Canada average wage in Ontario.^22^ Time spent providing assistance when the caregiver was not working, or did not take time off from work,^23^ was valued based on a proxy wage of a family caregiver in Ontario.^20^ Detailed unit costs and their sources^20,21,24,25,26,27,28,29,30,31,32,33^ are provided in eTables 2 to 7 in Supplement 1.

### Measure of Estimated Effectiveness

The primary measure of estimated effectiveness of this economic evaluation was the number of quality-adjusted life-years (QALYs).^16,34,35,36^ Quality-adjusted life-years combine quantity of life with quality of life, whereby quality of life is represented by a health utility score ranging from 0 (death) to 1 (full health). Using the Canadian algorithm,^37^ we derived health utility scores from the EQ-5D-5L instrument, which captures current health status (ie, as of today) in 5 domains (mobility, self-care, usual activities, pain/discomfort, and anxiety/depression). The EQ-5D-5L was administered at ICU discharge, hospital discharge, and postdischarge follow-up. Since patients were randomized in the ICU, a baseline utility value of 0 was used.^38^ We calculated QALYs by measuring the area under the curve between time points (baseline, ICU discharge, hospital discharge, follow-up) using utility scores and the time between time points, assuming a linear relationship between time points. Patients who died during the trial were assigned a zero-utility value at the time of death and beyond, and the date of death was used to calculate QALYs. This economic evaluation does not include any equity weighting of QALYs.

### Statistical Analysis

The statistical and cost-effectiveness analyses followed an intention-to-treat approach. We present descriptive statistics in frequencies and percentages or means and SDs. We imputed missing 90-day health care resource use data or EQ-5D-5L responses among hospital survivors (eg, vanguard patients and those enrolled after April 1, 2018, with missing data) using the multiple imputation approach described in the CYCLE report.^13^ Means and SEs pooled from imputed datasets were used for the base case analyses to determine the costs and QALYs for each group. In the absence of dominance (ie, 1 strategy is associated with higher costs and fewer QALYs than the alternative), an incremental cost per QALY gained was calculated to compare the 2 groups.^35^ To deal with data skewness, deal with sampling uncertainty, and preserve the correlation between costs and QALYs,^14,39^ we used bootstrap techniques to compare the per-patient costs and QALYs between the 2 groups using 1000 bootstraps, with each bootstrap pooling the results from each of the 100 imputations using Rubin rules.^40^ We generated 95% CIs around the cost and QALY differences and the incremental cost-effectiveness ratio using the percentile method. Cost-effectiveness acceptability curves^41^ were used to present the probability of the intervention being cost-effective at a willingness to pay of CA$50 000 per QALY gained, which is commonly used in Canada.^42^

Per the study protocol,^12,13^ we conducted subgroup analyses based on age (≥65 vs <65 years), sex (male vs female), and baseline Clinical Frailty Scale score (≥5 [with frailty] vs <5 [without frailty]). We also conducted several sensitivity analyses to explore the impact of varying key assumptions: (1) restricting the time horizon to 90 days after randomization for all patients (ie, calculating costs and QALYs at 90 days for patients who had their follow-up after 90 days post randomization); (2) varying all unit costs by plus or minus 30% of the base case values; and (3) comparing the 90-day cost and EQ-5D-5L utilities based on nonimputed and imputed data. All analyses were conducted using SAS, version 9.4 (SAS Institute Inc). Statistical significance was defined as *P* < .05.

## Results

**Figure 1.**
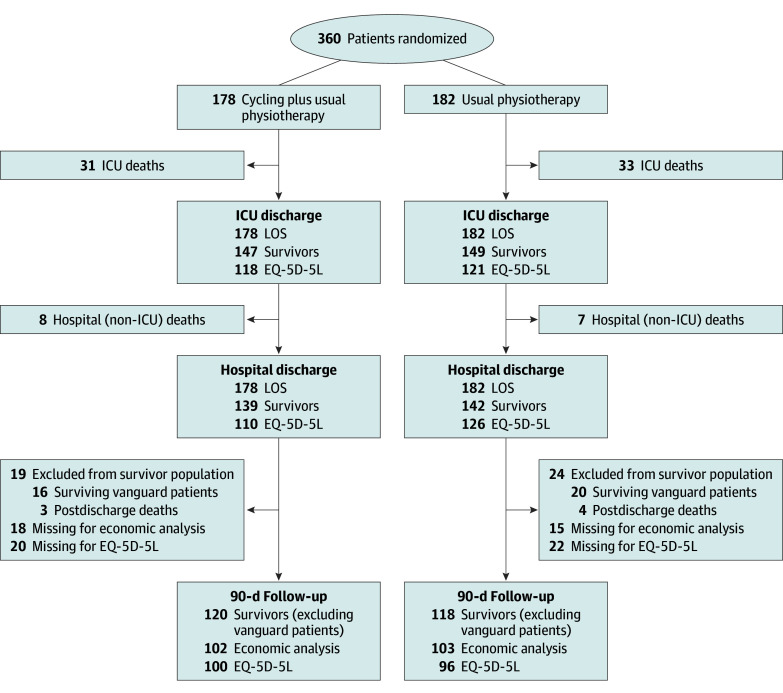
Number of Patients Contributing Data to the Cost-Effectiveness Analysis At each time point, the number of patients alive and the number contributing to the length of stay (LOS), economic, or health-related quality-of-life (EQ-5D-5L) analyses are reported. The Critical Care Cycling to Improve Lower Extremity Strength (CYCLE) trial included a vanguard phase of 46 patients for whom postrandomization 90-day follow-up data were not collected due to lack of funding. ICU indicates intensive care unit.

The number of patients contributing to the economic evaluation before imputation is shown in Figure 1. Among the 360 patients randomized in the CYCLE trial (178 in the cycling plus usual physiotherapy group, 182 in the usual physiotherapy alone group; mean [SD] age, 61.5 [15.6] years; 155 female [43.1%] and 205 male [56.9%]) in 13 medical-surgical ICUs across Canada, 2 in the US, and 1 in Australia, 281 (78.1%) survived the index hospitalization. Of the 281 survivors, 36 (12.8%) were enrolled in the vanguard phase and did not have 90-day follow-up data. From the remaining 245 patients alive at hospital discharge, 7 (2.9%) had died by the 90-day follow-up, leaving 238 patients (97.1%) available for 90-day follow-up. Among the 238 patients with a 90-day follow-up, the median follow-up time for cycling plus usual physiotherapy vs usual physiotherapy alone was 94 days (IQR, 91-105 days) and 95 days (IQR, 91-106 days), respectively. After hospital discharge, we obtained economic data for 205 patients (86.1%) and EQ-5D-5L data for 196 patients (82.3%). eTables 8 to 14 in Supplement 1 present the detailed 90-day economic data based on nonimputed data. In the following, all results are based on imputed data unless otherwise specified.

### Cost Analysis

Based on responses from 11 of 16 sites, approximately 37 patients were expected to use the in-bed cycle annually, which resulted in an equipment cost of CA$136 per patient (CA$25 000 per in-bed cycle divided by 5 years divided by 36.8 patients per year). The mean (SE) costs associated with the index hospitalization were CA$66 554 (CA$4301) for cycling plus usual physiotherapy and CA$61 935 (CA$5387) for usual physiotherapy alone for a difference of CA$4619 (95% CI, −CA$8868 to CA$17 748). This difference was attributed to the longer, but not statistically different index admission LOS associated with cycling plus usual physiotherapy (difference, 1.9 days; 95% CI, −4.9 to 8.1 days). The differences between the 2 groups regarding 90-day follow-up costs, total costs, or individual cost components were also not statistically different. The index hospitalization costs accounted for approximately 90% of the total costs. In contrast, the cycling costs per patient (CA$136 for the bike; mean [SE] cost for bike setup and takedown, CA$75 [CA$5]; mean [SE] cost for delivering cycling sessions, CA$110 [CA$8]) accounted for 0.5% of the index hospitalization costs (CA$321 of CA$66 554 per patient enrolled in the cycling plus usual physiotherapy group). The per-patient differences in 90-day costs were CA$5841 (95% CI, −CA$7666 to CA$18 797) (Table 1).

**Table 1.** Health Care Resource Use, Productivity Losses, and Costs

Variable	Mean (SE)				Difference in costs between cycling plus usual physiotherapy and usual physiotherapy alone, CA$ (95% CI)
	Cycling plus usual physiotherapy (n = 178)		Usual physiotherapy alone (n = 182)		
	Resource use	Costs, CA$	Resource use	Costs, CA$	
**Index hospitalization**					
Cost of the bike	NA	136	NA	NA	136
Bike setup, min	79.34 (5.26)	75 (5)	NA	NA	75 (65 to 86)
Cycling session, min^a^	116.1 (8.04)	110 (8)	NA	NA	110 (96 to 126)
Usual physiotherapy session, min^b^	124.29 (6.84)	154 (12)	155.08 (9.84)	200 (13)	−46 (−78 to −14)
ICU days from randomization	13.53 (0.99)	49 467 (3628)	12.66 (1.11)	46 271 (4052)	3196 (−6975 to 13 3384)
Non-ICU days from randomization	14.22 (1.83)	16 612 (2136)	13.24 (1.92)	15 464 (2234)	1148 (−4985 to 7223)
Subtotal 1	NA	66 554 (4301)	NA	61 935 (5387)	4619 (−8868 to 17 748)
**90-d Follow-up after randomization (data after hospital discharge)**					
ICU days	0.36 (0.21)	1338 (768)	0.10 (0.05)	363 (182)	974 (−74 to 2529)
Non-ICU days	1.47 (0.42)	1724 (489)	0.83 (0.36)	975 (419)	749 (−414 to 1930)
ED visits	0.27 (0.06)	99 (20)	0.22 (0.04)	78 (15)	21 (−19 to 63)
GP visits	0.57 (0.09)	22 (3)	0.60 (0.09)	23 (3)	−1 (−10 to 7)
Physician specialist visits	Varies	133 (17)	Varies	133 (19)	0 (−41 to 31)
Other HCP visits	Varies	50 (12)	Varies	54 (15)	−3 (−48 to 41)
Long-term-care days	0.52 (0.32)	150 (92)	0.65 (0.33)	189 (95)	−39 (−266 to 203)
Assisted living days	0.45 (0.44)	55 (55)	0.00 (0.00)	0 (0)	55 (3 to 157)
Chronic care facility days	0.24 (0.24)	287 (284)	0.27 (0.27)	319 (319)	−32 (−809 to 745)
Inpatient rehabilitation days	1.73 (0.50)	1938 (557)	1.57 (0.51)	1760 (570)	177 (−1223 to 1469)
Other facility days	0	0	0.95 (0.70)	355 (293)	−355 (−859 to −61)
Hours of assistance needed by others	24.63 (5.06)	506 (104)	32.94 (7.84)	671 (159)	−165 (−537 to 172)
Hours taken off from work for those assisting the patient	6.69 (4.77)	310 (221)	6.72 (2.33)	312 (110)	−2 (−392 to 507)
Hours of work taken off by the patient	37.19 (8.72)	1721 (404)	40.35 (9.23)	1867 (427)	−146 (−1038 to 879)
Subtotal 2	NA	8332 (1327)	NA	7111 (1037)	1221 (−1578 to 4054)
Total	NA	74 887 (4524)	NA	69 046 (5440)	5841 (−7666 to 18 797)

Abbreviations: CA$, Canadian dollars; ED, emergency department; GP, general practitioner; HCP, health care professional; ICU, intensive care unit; NA, not applicable.

^a^ The information on the minutes associated with the cycling plus usual physiotherapy sessions was based on the average total time across the patient’s ICU stay.

^b^ The costs associated with the sessions of usual physiotherapy took into account the proportion of time when 2 physiotherapists were present instead of 1 (30% for the cycling plus usual physiotherapy group and 36% for the usual physiotherapy alone group).

### Estimated Effectiveness

The mean (SE) utilities among survivors were not significantly different between cycling plus usual physiotherapy and usual physiotherapy alone at ICU discharge (0.51 [0.03] vs 0.52 [0.03]; difference, −0.01; 95% CI, −0.07 to 0.06), hospital discharge (0.71 [0.03] vs 0.67 [0.02]; difference, 0.04; 95% CI, −0.01 to 0.10), and at the 90-day follow-up (0.80 [0.02] vs 0.78 [0.02]; difference, 0.02; 95% CI, −0.02 to 0.07). The mean (SE) number of QALYs associated with cycling plus usual physiotherapy and usual physiotherapy alone were 0.1396 (0.0069) and 0.1404 (0.0071), respectively, for a nonstatistically significant difference of −0.0009 (95% CI, −0.0185 to 0.0182).

### Cost-Effectiveness Analyses

**Figure 2.**
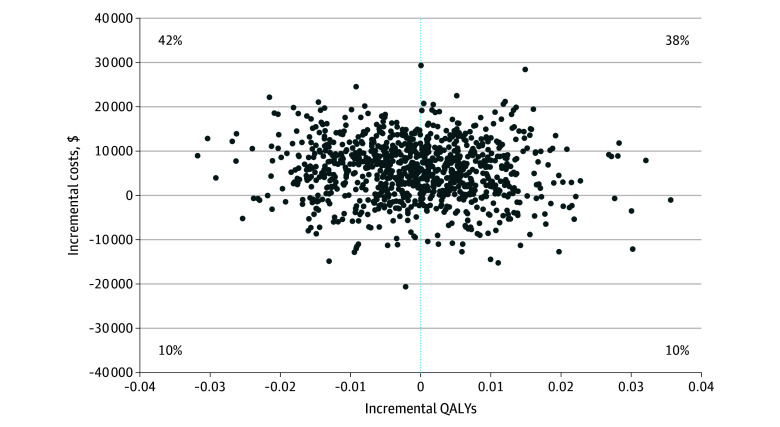
Incremental Costs and Quality-Adjusted Life-Years (QALYs) of Cycling Plus Usual Physiotherapy vs Usual Physiotherapy Alone This scatter plot of 1000 bootstrap simulations summarizes the costs and QALYs of early intensive care unit cycling plus usual physiotherapy vs usual physiotherapy alone.

**Figure 3.**
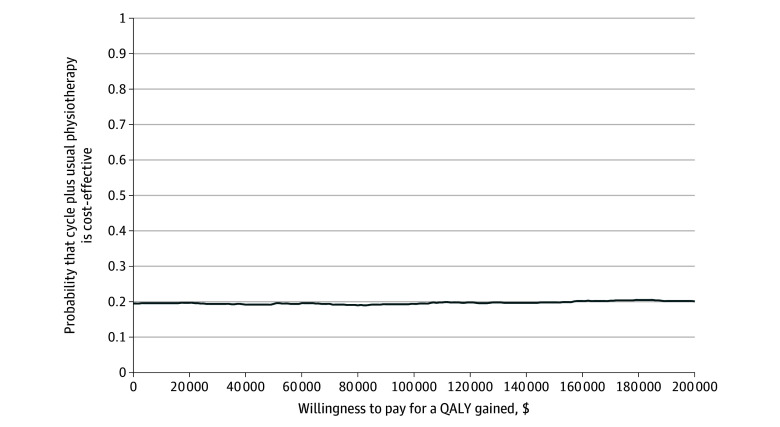
Cost-Effectiveness Acceptability Curve QALY indicates quality-adjusted life-year.

Cycling plus usual physiotherapy was dominated by usual physiotherapy alone (higher costs and fewer QALYs) in 42% of the bootstrap resamples (Figure 2). Since the bootstrap resamples spanned across all 4 quadrants of the cost-effectiveness plane, the 95% CIs for the incremental cost-effectiveness ratio were undefined. As shown by the cost-effectiveness acceptability curve presented in Figure 3, the probability of cycling plus usual physiotherapy being cost-effective was 0.19 at a willingness to pay threshold of CA$50 000 per QALY gained.

### Subgroup and Sensitivity Analyses

Although there were some differences between subgroups and among our sensitivity analyses, the costs and QALYs were not significantly different from 0 (Table 2). eTables 15 to 17 in Supplement 1 (health care resource use in general, for specialist visits, and for other health care professional visits used for the costing) and eTable 18 in Supplement 1 (90-day costs and QALYs) present the 90-day follow-up results from nonmissing or imputed data.

**Table 2.** Subgroup and Sensitivity Analyses

Variable	Mean (SE)		Difference between cycling plus usual physiotherapy and usual physiotherapy alone (95% CI)	Incremental cost per QALY gained (and 95% CI) comparing cycling plus usual physiotherapy with usual physiotherapy alone^a^^,^^b^
	Cycling plus usual physiotherapy (n = 178)	Usual physiotherapy alone (n = 182)		
**Main analysis**				
Costs, CA$	74 887 (4524)	69 046 (5440)	5841 (−7666 to 18 797)	Cycling plus usual physiotherapy is dominated by usual physiotherapy alone (higher costs, fewer QALYs)^c^
QALYs	0.1396 (0.0069)	0.1404 (0.0071)	−0.0009 (−0.0185 to 0.0182)	
**Prespecified subgroup analyses**				
Age <65 y (n = 181)				
Costs, CA$	75 563 (6361)	73 164 (9371)	2399 (−23 220 to 22 561)	CA$8 387 386/QALY gained (ie, CA$2398.7923/0.000286)^b^^,^^c^
QALYs	0.1511 (0.0090)	0.1508 (0.0094)	0.0003 (−0.0225 to 0.0263)	
Age ≥65 y (n = 179)				
Costs, CA$	74 226 (6455)	64 744 (5305)	9482 (−6536 to 25 907)	Cycling plus usual physiotherapy is dominated by usual physiotherapy alone (higher costs, fewer QALYs)^c^
QALYs	0.1283 (0.0104)	0.1296 (0.0105)	−0.0014 (−0.0327 to 0.0278)	
Prehospital Clinical Frailty Scale score <5 (n = 307)				
Costs, CA$	73 943 (4637)	64 786 (4184)	9157 (−916 to 24 422)	Cycling plus usual physiotherapy is dominated by usual physiotherapy alone (higher costs, fewer QALYs)^c^
QALYs	0.1414 (0.0076)	0.1482 (0.0078)	−0.0068 (−0.0282 to 0.0126)	
Prehospital Clinical Frailty Scale score ≥5 (n = 53)				
Costs, CA$	80 407 (15 154)	106 817 (26 992)	−26 410 (−88 353 to 24 574)	Cycling plus usual physiotherapy dominates usual physiotherapy alone (lower costs, higher QALYs)^c^
QALYs	0.1290 (0.0175)	0.0960 (0.0146)	0.0331 (−0.0095 to 0.0768)	
Male sex (n = 205)				
Costs, CA$	73 319 (5460)	64 786 (5555)	8533 (−5980 to 24 311)	Cycling plus usual physiotherapy is dominated by usual physiotherapy alone (higher costs, fewer QALYs)^c^
QALYs	0.1346 (0.0094)	0.1425 (0.0091)	−0.0078 (−0.0324 to 0.0160)	
Female sex (n = 155)				
Costs, CA$	77 041 (7704)	74 479 (10 162)	2562 (−20 292 to 24 819)	CA$303 027/QALY gained (CA$2561.4892/0.008448)^b^^,^^c^
QALYs	0.1463 (0.0101)	0.1379 (0.0113)	0.0084 (−0.0197 to 0.0361)	
**Sensitivity analyses**				
Follow-up limited to 90-d after randomization				
Costs, CA$	68 180 (3941)	61 205 (3694)	6975 (−3500 to 16 912)	CA$7 944 482/QALY gained (CA$6975.2641/0.000878)^b^^,^^c^
QALYs	0.1230 (0.0060)	0.1221 (0.0058)	0.0009 (−0.0147 to 0.0167)	
Unit costs increased by 30% (except for physician fees)				
Costs, CA$	96 133 (5811)	88 640 (6985)	7493 (−11 075 to 25 250)	Cycling plus usual physiotherapy is dominated by usual physiotherapy alone (higher costs, fewer QALYs)^c^
QALYs	0.1396 (0.0069)	0.1404 (0.0071)	−0.0009 (−0.0185 to 0.0182)	
Unit costs decreased by 30% (except for physician fees)				
Costs, CA$	53.640 (3238)	49 453 (3896)	4187 (−5637 to 14 503)	Cycling plus usual physiotherapy is dominated by usual physiotherapy alone (higher costs, fewer QALYs)^c^
QALYs	0.1396 (0.0069)	0.1404 (0.0071)	−0.0009 (−0.0185 to 0.0182)	

Abbreviations: CA$, Canadian dollars; QALY, quality-adjusted life-year.

^a^ This table describes the 3 prespecified subgroup and 3 sensitivity analyses for the economic evaluation. The incremental cost-effectiveness ratio per QALY gained is based on point estimates when cycling plus usual physiotherapy is compared with usual physiotherapy alone. Cycling plus usual physiotherapy dominates usual physiotherapy alone when cycling plus usual physiotherapy is less expensive and offers more QALYs than usual physiotherapy alone. Cycling plus usual physiotherapy is dominated by usual physiotherapy alone when cycling plus usual physiotherapy is more expensive and offers fewer QALYs than usual physiotherapy alone. In the absence of dominance, the incremental cost-effectiveness ratio per QALY is calculated as the ratio of the difference in costs and the difference in QALYs when cycling plus usual physiotherapy is compared with usual physiotherapy alone.

^b^ In an effort to minimize rounding errors in the calculation of the incremental cost-effectiveness ratios per QALY gained, the incremental cost and QALY data presented in the parentheses include 4 and 6 decimals, respectively.

^c^ The 95% CIs around the incremental cost per QALY gained could not be defined as the bootstrap resamples spanned across all 4 quadrants of the cost-effectiveness plane.

## Discussion

In this trial-based economic evaluation, cycling added to usual physiotherapy for adults receiving mechanical ventilation in the ICU was associated with a marginal increase in the index hospitalization cost (ie, 0.5% of the actual index hospitalization costs). We did not find any significant between-group differences in terms of 90-day costs and QALYs in our main, subgroup, or sensitivity analyses. The results align with the CYCLE trial,^13^ which did not show a difference between groups for the primary study outcome (Physical Function ICU Test score at 3 days after ICU discharge) or other secondary outcomes (eg, ICU and total LOS).

Although there was no difference in the primary study outcome, we conducted an economic evaluation of the CYCLE trial because many Canadian funding agencies require an economic evaluation alongside clinical trials to inform the implementation of the trial results into practice. In addition, because absence of evidence is not evidence of absence, several health economists,^41,43,44^ economic guidelines,^14,35^ and a recent editorial^45^ recommended joint comparisons of costs and outcomes, even if a trial does not show any statistically significant differences in clinical outcomes.

Despite the role of trial-based economic evaluations to inform decision-making, reliance on a single RCT provides an incomplete picture of the consequences of an intervention; this needs to be interpreted in light of the overall body of evidence, such as a systematic review. While the CYCLE trial showed no difference in LOS between the groups, a recent systematic review and meta-analysis^11^ of 33 RCTs (including CYCLE) that compared cycling interventions with a no cycling intervention among critically ill adults admitted to the ICU for longer than 24 hours found that cycling may decrease ICU LOS (29 RCTs; 2575 patients; 1.06 fewer days; 95% CI, 0.33-1.80 fewer days; low certainty) and probably decreases hospital LOS (22 RCTs; 2060 patients; 1.48 fewer days; 95% CI, 0.47-2.49 fewer days; moderate certainty). Future research is needed to evaluate the cost-effectiveness of cycling interventions based on the totality of RCT evidence.

This economic evaluation of the CYCLE trial contributes to critical care and rehabilitation by acknowledging the importance of understanding health resource implications, productivity losses, and health-related quality of life after hospital discharge. Due to the increased role of cost-effectiveness analysis to support funding allocations in the context of scarce health care resources, quality-of-life and economic information are important to collect alongside RCTs to inform decision-making. We are aware of one other trial-based economic evaluation of early rehabilitation for patients receiving ventilation. This trial collected costs and quality-of-life data up to 180 days after hospital discharge.^46^ Results were similar to ours, showing no differences in costs and QALYs between high-dose early active mobilization and usual care mobilization.

### Strengths and Limitations

This economic evaluation had several strengths. It occurred alongside the CYCLE RCT wherein patient-reported EQ-5D-5L quality-of-life data and actual costs were collected. CYCLE was partially embedded in the health care system at participating sites, in which frontline physiotherapists recorded direct treatment duration. To measure the outcomes of the interventions, patients completed 90-day follow-up assessments after hospital discharge. High survivor retention documented health care resource use, assistance received from others, and productivity losses.

Several limitations were also associated with this economic evaluation. Not all patients contributed to the 90-day follow-up, and missing data were imputed.^14^ We used unit costs from Ontario to value health care resource use. The hospital costing data were based on patient-level costing data for 63 patients recruited from 3 hospitals in Hamilton, Ontario; however, these patients were not different from the others. Follow-up health care use was based on patient reports and may be subject to recall bias. Alternative in-bed cycle models than the one used in the study may have different prices. To address these limitations, we conducted several sensitivity analyses (eg, changes in ICU and non-ICU unit costs), for which the results were consistent with the main analysis, thus increasing our confidence in our findings. Finally, since the level of usual physiotherapy in the CYCLE RCT was among the highest across all rehabilitation RCTs,^11^ the results may not be generalizable to centers without similar levels of usual physiotherapy (approximately 30 minutes per day).

## Conclusions

In this trial-based economic evaluation, the differences in costs and QALYs between adding in-bed cycling to usual physiotherapy and usual physiotherapy alone for adults undergoing mechanical ventilation were not significantly different from 0. These results highlight the need for additional cost-effectiveness studies considering the full body of evidence regarding in-bed cycling for critically ill patients.

## Notes

Supplement 1. **eTable 1.** Baseline Characteristics of Patients (n = 63) Included in the Estimation of Daily ICU and Non-ICU Costs vs Those Not Included (n = 297) **eTable 2.** Unit Costs, Rounded to the Nearest Dollar (2024 Canadian Dollars) **eTable 3.** Cost Per Physician Specialist Visits, Rounded to the Nearest Dollar (2024 Canadian Dollars) **eTable 4.** List of “Other” Physician Visits and Unit Costs for Cycling Plus Usual Physiotherapy, Rounded to the Nearest Dollar (2024 Canadian Dollars) **eTable 5.** List of “Other” Specialties and Unit Costs for Usual Physiotherapy, Rounded to the Nearest Dollar (2024 Canadian Dollars) **eTable 6.** Unit Costs of Health Care Professionals and Unit Costs, Rounded to the Nearest Dollar (2024 Canadian Dollars) **eTable 7.** List of “Other” Health Care Professionals and Unit Costs, Rounded to the Nearest Dollar (2024 Canadian Dollars) **eTable 8.** 90-Day Follow-Up Questionnaire: Hospitalization and Emergency Department Visits (Nonimputed Data) **eTable 9.** 90-Day Follow-Up Questionnaire: Physician Visits (Nonimputed Data) **eTable 10.** 90-Day Follow-Up Questionnaire: Other Health Care Professionals (Nonimputed Data) **eTable 11.** 90-Day Follow-Up Questionnaire: Days Spent in Facilities (Nonimputed Data) **eTable 12.** Admission to Other Health Care Facilities Based on Living Status Before Index Admission: Cycling Plus Usual Physiotherapy (Nonimputed Data) **eTable 13.** Admission to Other Health Care Facilities Based on Living Status Before Index Admission: Usual Physiotherapy (Nonimputed Data) **eTable 14.** Assistance From Others and Work Time Lost (Nonimputed Data) **eTable 15.** Comparison of 90-Day Nonimputed and Imputed Health Care Resource Use **eTable 16.** Comparison of Nonimputed Case and Imputed Health Care Resource Use: Specialist Visits **eTable 17.** Comparison of Nonimputed and Imputed Health Care Resource Use: Other Health Care Professional Visits **eTable 18.** Comparison of Nonimputed and Imputed 90-Day Follow-Up Costs and 90-Day EQ-5D-5L
eReferences.

Supplement 2.
Group Information

Supplement 3.
Data Sharing Statement
