# Supplementary material for: Cost-Effectiveness of In-Bed Cycling and Routine Physiotherapy for Patients Receiving Mechanical Ventilation
Source: JAMA Netw Open. 2025 Sep 8;8(9):e2529399. doi: 10.1001/jamanetworkopen.2025.29399 (PMC12418132; doi:10.1001/jamanetworkopen.2025.29399)
Supplement: Supplement 1. — eTable 1. Baseline Characteristics of Patients (n = 63) Included in the Estimation of Daily ICU and Non-ICU Costs vs Those Not Included (n = 297) eTable 2. Unit Costs, Rounded to the Nearest Dollar (2024 Canadian Dollars) eTable 3. Cost Per Physician Specialist Visits, Rounded to the Nearest Dollar (2024 Canadian Dollars) eTable 4. List of “Other” Physician Visits and Unit Costs for Cycling Plus Usual Physiotherapy, Rounded to the Nearest Dollar (2024 Canadian Dollars) eTable 5. List of “Other” Specialties and Unit Costs for Usual Physiotherapy, Rounded to the Nearest Dollar (2024 Canadian Dollars) eTable 6. Unit Costs of Health Care Professionals and Unit Costs, Rounded to the Nearest Dollar (2024 Canadian Dollars) eTable 7. List of “Other” Health Care Professionals and Unit Costs, Rounded to the Nearest Dollar (2024 Canadian Dollars) eTable 8. 90-Day Follow-Up Questionnaire: Hospitalization and Emergency Department Visits (Nonimputed Data) eTable 9. 90-Day Follow-Up Questionnaire: Physician Visits (Nonimputed Data) eTable 10. 90-Day Follow-Up Questionnaire: Other Health Care Professionals (Nonimputed Data) eTable 11. 90-Day Follow-Up Questionnaire: Days Spent in Facilities (Nonimputed Data) eTable 12. Admission to Other Health Care Facilities Based on Living Status Before Index Admission: Cycling Plus Usual Physiotherapy (Nonimputed Data) eTable 13. Admission to Other Health Care Facilities Based on Living Status Before Index Admission: Usual Physiotherapy (Nonimputed Data) eTable 14. Assistance From Others and Work Time Lost (Nonimputed Data) eTable 15. Comparison of 90-Day Nonimputed and Imputed Health Care Resource Use eTable 16. Comparison of Nonimputed Case and Imputed Health Care Resource Use: Specialist Visits eTable 17. Comparison of Nonimputed and Imputed Health Care Resource Use: Other Health Care Professional Visits eTable 18. Comparison of Nonimputed and Imputed 90-Day Follow-Up Costs and 90-Day EQ-5D-5L eReferences. [file jamanetwopen-e2529399-s001.pdf]

Supplemental Online Content

Tarride J-E, Blackhouse G, Rochwerg B, et al. In-bed cycling and routine physiotherapy for patients receiving mechanical ventilation. *JAMA Netw Open*. 2025;8(8):e2529399. doi:10.1001/jamanetworkopen.2025.29399

eTable 1. Baseline Characteristics of Patients (n = 63) Included in the Estimation of Daily ICU and Non-ICU Costs vs Those Not Included (n = 297)

eTable 2. Unit Costs, Rounded to the Nearest Dollar (2024 Canadian Dollars)

eTable 3. Cost Per Physician Specialist Visits, Rounded to the Nearest Dollar (2024 Canadian Dollars)

eTable 4. List of "Other" Physician Visits and Unit Costs for Cycling Plus Usual Physiotherapy, Rounded to the Nearest Dollar (2024 Canadian Dollars)

eTable 5. List of "Other" Specialties and Unit Costs for Usual Physiotherapy, Rounded to the Nearest Dollar (2024 Canadian Dollars)

eTable 6. Unit Costs of Health Care Professionals and Unit Costs, Rounded to the Nearest Dollar (2024 Canadian Dollars)

eTable 7. List of "Other" Health Care Professionals and Unit Costs, Rounded to the Nearest Dollar (2024 Canadian Dollars)

eTable 8. 90-Day Follow-Up Questionnaire: Hospitalization and Emergency Department Visits (Nonimputed Data)

eTable 9. 90-Day Follow-Up Questionnaire: Physician Visits (Nonimputed Data)

eTable 10. 90-Day Follow-Up Questionnaire: Other Health Care Professionals (Nonimputed Data)

eTable 11. 90-Day Follow-Up Questionnaire: Days Spent in Facilities (Nonimputed Data)

eTable 12. Admission to Other Health Care Facilities Based on Living Status Before Index Admission: Cycling Plus Usual Physiotherapy (Nonimputed Data)

eTable 13. Admission to Other Health Care Facilities Based on Living Status Before Index Admission: Usual Physiotherapy (Nonimputed Data)

eTable 14. Assistance From Others and Work Time Lost (Nonimputed Data)

eTable 15. Comparison of 90-Day Nonimputed and Imputed Health Care Resource Use

eTable 16. Comparison of Nonimputed Case and Imputed Health Care Resource Use: Specialist Visits

eTable 17. Comparison of Nonimputed and Imputed Health Care Resource Use: Other Health Care Professional Visits

eTable 18. Comparison of Nonimputed and Imputed 90-Day Follow-Up Costs and 90-Day EQ-5D-5L

eReferences

© 2025 Tarride J-E et al. *JAMA Network Open*.

This supplemental material has been provided by the authors to give readers additional information about their work.

eTable 1. Baseline Characteristics of Patients (n = 63) Included in the Estimation of Daily ICU and Non-ICU Costs vs Those Not Included (n = 297)

	Hospital Cost Cohort (n=63)	Cohort without Hospital Costs (n=297)	p-value
**Baseline Characteristics**			
Age - mean (SD)	64.0 (13.0)	60.9 (16.1)	0.14
Female - n (%)	25 (39.7%)	130 (33.9%)	0.48
APACHE II - mean (SD)	25.6 (8.3)	23.6 (8.5)	0.09
Frailty and Function			
Pre-hospital-independent and at home- n (%)	52 (82.5%)	261 (88.8%)	0.14
Pre-hospital frailty score - mean (SD)	3.2 (1.3)	3.2 (1.3)	0.82
Charlson comorbidity index - mean (SD)	2.7 (2.2)	1.6 (1.8)	<0.01
Functional comorbidity index - mean (SD)	2.2 (1.7)	1.8 (1.5)	0.14
Katz Activity of daily living index - mean (SD)	5.9 (0.4)	5.8 (0.8)	0.45
ICU admission type - n (%)			0.22
Medical	60 (95.2%)	262 (89.1%)	
Surgical	3 (4.8%)	35 (11.9%)	
ICU admission Diagnosis - n (%)			0.03
Respiratory	28 (44.4%)	131 (44.1%)	0.88
Gastrointestinal	6 (9.5%)	40 (13.5%)	0.37
Sepsis	16 (25.4%)	30 (10.1%)	0.00
Cardiovascular/vascular	6 (9.5%)	26 (8.8%)	0.84
Neurologic	2 (3.2%)	22 (7.4%)	0.21
Metabolic	2 (3.2%)	17 (5.7%)	0.40
Other	3 (4.8%)	31 (10.4%)	0.15
Interventions at randomization			
Mechanical ventilation - n (%)	63 (100.0%)	297 (100%)	
Inotropes or vasopressors - n (%)	28 (44.4%)	176 (59.3%)	0.02
Renal replacement therapy - n (%)	10 (15.9%)	34 (11.4%)	0.36
Length of stay - mean (SD) days			
ICU	16.0 (15.0)	12.5 (13.9)	0.06
Hospital	31.0 (24.2)	25.9 (33.1)	0.25

Legend for eTable1: This table compares the patient characteristics of the costing cohort of 63 patients from 3 hospitals in Hamilton, ON, Canada and the remaining cohort.
SD: standard deviation

© 2025 Tarride J-E et al. *JAMA Network Open*.

eTable 2. Unit Costs, Rounded to the Nearest Dollar (2024 Canadian Dollars)

Cost items	Unit Costs	Source
**Index hospitalization**		
Intensive care unit (ICU) per day	$3,426	Estimated from patient level data on 63 patients enrolled in 3 hospitals, Hamilton, Ontario, Canada
ICU physician Day 1	$383	Ontario Schedule of Benefits 2024 (code G557)[1]
ICU physician Day 2 to 30 inclusive	$229	Ontario Schedule of Benefits 2024 (code G558)[1]
ICU physician Day 31 and beyond.	$116	Ontario Schedule of Benefits 2024 (code G559)[1]
General ward per day	$1,120	Estimated from patient level data on 63 patients enrolled in 3 hospitals from Hamilton, Ontario, Canada
Most responsible physician Day 1	$61	Ontario Schedule of Benefits 2024 (C122)[1]
Most Responsible physician Day 2	$61	Ontario Schedule of Benefits (C123)[1]
Most Responsible Physician fee bonus	30% added to C122 and C123	Ontario Schedule of Benefits (E083)[1]
Most Responsible physician Day 3 and beyond	$34	Ontario Schedule of Benefits (C121)[1]
Specialized in-bed cycle ergometer per Patient	$136	Assumes that each bike costs $25,000 and is amortized over 5 years (Cycle RCT Protocol[2]) and that the bike will be used by an average of 37 patients per year based on the responses of participating sites.
Physiotherapist per hour (including 30% benefits)	$57	Job Bank Canada - Ontario[3]
**90-day follow-up**		
ICU day	$3,493	Estimated from patient level data on 63 patients enrolled in 3 hospitals, Hamilton, Ontario, Canada. In the absence of costing data for re-hospitalizations, for 90-day follow-up, we assumed the same ICU cost for the index hospitalization, and added the cost associated with the 1st 2 days in the ICU. We did not include these costs for the initial hospitalization, because randomization occurred post ICU admission.
General ward per day	$1,120	Estimated from patient level data on 63 patients enrolled in 3 hospitals from Hamilton, Ontario, Canada. In the absence of costing data for re-hospitalizations, assumed to be the same as the general ward cost for the index hospitalization.
Emergency department visit	$355	Canadian Institute for Health Information[4]
General practitioner	$38	Ontario Schedule of Benefits 2024 (code: A004)[1]
Specialist physician	Varies	Varies from $94 to $325 (see eTable 3)
Other health care professionals	Varies	Varies from $32 to $101 (see eTable 6)
Retirement home Per day	$123	Canada Mortgage and Housing Corporation ($3,354 per month)[5]
Assisted living per day	$123	Assumed to be same as daily cost of retirement home. Canada Mortgage and Housing Corporation ($3,354 per month)[5]

© 2025 Tarride J-E et al. *JAMA Network Open*.

Cost items	Unit Costs	Source
Long term care per day	$289	Government of Ontario (daily cost of co-pay of $80.72 + cost of government for personal care of 208.65)[6]
Chronic care facility per day	$1,187	Government of Ontario (cost per non-ICU day of $1,120 + daily copay of $66.95)[7]
Inpatient rehabilitation per day	$1,120	Assumed same as non-ICU day
Drug rehabilitation facility per day	$441	Addiction Rehab Toronto[8] https://addictionrehabtoronto.ca/how-much-does-rehab-cost-in-ontario/
Outpatient psychiatric hospital per day	$210	Daily cost per session of cognitive behavioral therapy.[9]
Hourly wage in Ontario for individuals aged 55 years or more	$46	Statistics Canada[10] ($36 and 30% benefits)
Hourly wage for caregiver not working or taking time off work to provide assistance	$20	Job Bank Canada- Ontario (category: family caregiver)[3]

Notations: ICU: intensive care unit; ED: emergency department.

© 2025 Tarride J-E et al. *JAMA Network Open*.

eTable 3. Cost Per Physician Specialist Visits, Rounded to the Nearest Dollar (2024 Canadian Dollars)

Type of specialty	Unit Costs	Source	Fee Code
Cardiologist	$162	Ontario Schedule of Benefits 2024[1]	A605
Dentist	$325	Willow Dental, Toronto[11]	-
Dermatologist	$72	Ontario Schedule of Benefits 2024[1]	A025
Ear, Nose & Throat	$84	Ontario Schedule of Benefits 2024[1]	A245
Gastroenterologist	$157	Ontario Schedule of Benefits 2024[1]	A415
Nephrologist	$163	Ontario Schedule of Benefits 2024[1]	A165
Neurologist	$184	Ontario Schedule of Benefits 2024[1]	A185
Psychiatrist	$222	Ontario Schedule of Benefits 2024[1]	A195
Physiatrist	$209	Ontario Schedule of Benefits 2024[1]	C315
Respirologist	$176	Ontario Schedule of Benefits 2024[1]	A475
Surgeon	$94	Ontario Schedule of Benefits 2024[1]	A095
Other (Cycling + Usual physiotherapy)	$142	Ontario Schedule of Benefits 2024[1]	Varies (see eTable 4 for details)
Other (Usual physiotherapy)	$151	Ontario Schedule of Benefits 2024[1]	Varies (see eTable 5 for details)

© 2025 Tarride J-E et al. *JAMA Network Open*.

eTable 4. List of "Other" Physician Visits and Unit Costs for Cycling Plus Usual Physiotherapy, Rounded to the Nearest Dollar (2024 Canadian Dollars)

Other physician visits	Unit costs (A)	Fee code	Frequency (n)	Percentage (B)	Weighted cost (A*B)
Allergy specialist	$38	A927	1	4%	$1
Endocrinologist	$165	A155	3	11%	$18
Epilepsy specialist	$184	A185	1	4%	$7
Haematologist	$172	A615	5	18%	$31
Infectious disease specialist	$182	A465	2	7%	$13
Internal medicine physician	$165	A135	3	11%	$18
Liver transplant surgeon	$138	n.a. (unit costs based on average of all "others")	1	4%	$5
Oncologist	$166	A445	4	14%	$24
Ophthalmologist	$82	A235	4	14%	$12
Radiologist	$50	A335	1	4%	$2
Rheumatologist	$178	A485	1	4%	$6
Thoracic surgeon	$99	A645	1	4%	$4
Urologist	$85	A355	1	4%	$3
**Total weighted cost**					**$142**

Notation: n.a.: not available

© 2025 Tarride J-E et al. *JAMA Network Open*.

eTable 5. List of “Other” Specialties and Unit Costs for Usual Physiotherapy, Rounded to the Nearest Dollar (2024 Canadian Dollars)

“Other” physician visits	Unit costs (A)	Fee code	Frequency (n)	Percentage (B)	Weighted cost (A*B)
Endocrinologist	$165	A155	2	6%	$10
Geriatrician	$203	A075	3	9%	$18
Hematologist	$172	A615	3	9%	$16
Infectious disease specialist	$182	A465	3	9%	$17
Internal medicine physician	$165	A135	4	12%	$20
Liver transplant surgeon	&140	n.a. (unit costs based on average of all “others”)	1	3%	$4
Oncologist	$167	A445	6	18%	$30
Ophthalmologist	$82	A235	2	6%	$5
Pain specialist	$38	A937	1	3%	$1
Plastic surgeon	$91	W085	1	3%	$3
Rheumatologist	$178	A485	1	3%	$5
Thoracic surgeon	$99	A645	3	9%	$9
Thrombosis specialist	$200	A384	1	3%	$6
Urologist	$85	A355	1	3%	$3
Wound specialist	$140	n.a. (unit costs based on average of all “others”)	1	3%	$4
**Total weighted cost**					$151

Notation: n.a.: not available

© 2025 Tarride J-E et al. *JAMA Network Open*.

eTable 6. Unit Costs of Health Care Professionals and Unit Costs, Rounded to the Nearest Dollar (2024 Canadian Dollars)

Type of other healthcare providers	Unit Costs	Source
Chiropractor[a]	$101	Ontario Chiropractor Assocation[12]
Dietician [a]	$51	Job Bank Canada - Ontario[3]
Personal Support Worker [a]	$27	Job Bank Canada - Ontario[3]
Meals on Wheels	$17	Meals on Wheels (cost per meal *2)[13]
Nurse practitioner [a]	$72	Job Bank Canada- Ontario[3]
Occupational Therapist [a]	$58	Job Bank Canada- Ontario[3]
Private Nurse [a]	$51	Job Bank Canada- Ontario[3]
Psychologist [a]	$51	Job Bank Canada- Ontario[3]
Physiotherapist [a]	$57	Job Bank Canada- Ontario[3]
Respiratory therapist [a]	$53	Job Bank Canada- Ontario[3]
Speech language Therapist [a]	$58	Job Bank Canada- Ontario[3]
Social Worker [a]	$49	Job Bank Canada- Ontario[3]
Transportation Services	$43	Bay Observer[14]
Visiting Nurse [a]	$51	Job Bank Canada- Ontario[3]
Other healthcare providers (Patients randomized to Cycling + Usual physiotherapy) [a]	$54	Job Bank Canada- Ontario[3]- See eTable 7 for details
Other healthcare providers (Patients randomized to Usual physiotherapy) [a]	$32	Job Bank Canada- Ontario[3] - See eTable 7 for details

[a]: include 30% benefits in addition to hourly wage.

© 2025 Tarride J-E et al. *JAMA Network Open*.

eTable 7. List of "Other" Health Care Professionals and Unit Costs, Rounded to the Nearest Dollar (2024 Canadian Dollars)

Other healthcare providers	Unit costs	Frequency(n)	Percentage	Weighted costs
**Cycling + Usual physiotherapy** [a]				
Emergency technologist	$53	1	33%	$18
Pharmacist	$67	1	33%	$22
Plastic technician	$42	1	33%	$14
**Total weighted cost**				**$54**
**Usual physiotherapy** [a]				
Acupuncturist	$26	1	17%	$4
Diabetes specialist	$51	1	17%	$9
Energy healer	$26	1	17%	$4
Kinesiologist	$34	1	17%	$6
Osteopath	$26	2	33%	$9
**Total weighted cost**				**$32**

[a]: include 30% benefits in addition to hourly wage. Source: Job Bank Canada- Ontario[3]

© 2025 Tarride J-E et al. *JAMA Network Open*.

eTable 8. 90-Day Follow-Up Questionnaire: Hospitalization and Emergency Department Visits (Nonimputed Data)

	Cycling + Usual physiotherapy n=178	Usual physiotherapy n=182
Number of ICU survivors at hospital discharge, n (%)	139 (78.1)	142 (78.0)
CYCLE Vanguard (no 90-day follow up), n (%)	16 (11.5)	20 (14.1)
Number of patients dying following hospital discharge	3 (1.7%)	4 (2.2%)
CYCLE (opportunity for 90-day follow up), n (%)	120 (86.3)	118 (83.1)
Number of survivors with follow-up opportunity at 90 days	120	118
Number of respondents (Economic Evaluation), n (%)	102 (85%)	103 (87%)
Number of patients responding whether or not they had a hospitalization post discharge, n (%)	101 (82.1)	103 (84.4)
Number of patients reporting any hospitalization, n (%)	21/101 (20.8%)	15/103 (14.6%)
• Mean (SD) number of hospitalizations among those reporting at least one hospitalization	1.4 (0.7)	1.2 (0.4)
Number of patients responding whether or not they had an ICU stay, n (%)	19/21 (90.5%)	13/15 (86.7%)
• Number of patients responding that they had an ICU stay, n (%)	4/19 (21.1%)	4/13 (30.8%)
• Mean (SD) ICU days among those responding that they had an ICU stay	11.0 (11.6)	8.5 (13.7)
Number of patients reporting a hospital stay without an ICU admission, n (%)	15/19 (78.9%)	9/13 (69.2%)
• Mean (SD) non-ICU days among those reporting non-ICU stays	11.0 (11.6)	8.5 (13.7)
Number of patients responding whether or not they had an emergency department visit post discharge, n (%)	95/101 (79.1)	93/103 (78.8)
Number of patients reporting that they had an ED visit, n (%)	25/95 (20.8%)	27/93 (22.9%)
• Number of patients reporting the number of visits, n (%)	24/25 (96%)	27/27 (100.0%)
• Mean (SD) number of ED visits among those indicating they had a visit	1.6 (0.9)	1.2 (0.6)

Notations: ICU: intensive care unit; LOS: length of stay; SD: standard deviation; ED: emergency department.

© 2025 Tarride J-E et al. *JAMA Network Open*.

eTable 9. 90-Day Follow-Up Questionnaire: Physician Visits (Nonimputed Data)

Physician Visits	Cycling + Usual physiotherapy (101/102 patients responded whether or not they had seen a physician since hospital discharge)			Usual physiotherapy (101/103 patients responded whether or not they had seen a physician since hospital discharge)		
	Number of patients reporting a visit	Number of patients providing the number of visits	Mean (SD) number of visits among those reporting a visit	Number of patients reporting a visit	Number of patients providing the number of visits	Mean (SD) number of visits among those reporting a visit
Family Doctor	46/101	42/46	1.9 (1.4)	51/101	49/51	1.8 (1.3)
Cardiologist	10/101	9/10	1.3 (0.5)	14/101	14/14	1.1 (0.4)
Dentist	5/101	5/5	1.0 (0.0)	2/101	2/2	1.0 (0.0)
Dermatologist	0/101	0	n.a.	2/101	2/2	1.0 (0.0)
Ear, Nose & Throat	4/101	4/4	1.3 (0.5)	1/101	1/1	3.0
Gastroenterologist	7/101	7/7	1.3 (0.5)	5/101	5/5	1.2 (0.4)
Nephrologist	9/101	7/9	1.1 (0.4)	3/101	3/3	1.3 (0.6)
Neurologist	2/101	2/2	2.0 (1.4)	1/101	1/1	1.0
Psychiatrist	1/101	0/1	n.a.	3/101	2/3	1.0 (0.0)
Physiatrist	1/101	1/1	1.0	1/101	0/1	n.a.
Respirologist	15/101	15/15	1.4 (0.9)	14/101	13/14	1.3 (0.5)
Surgeon	7/101	7/7	1.9 (1.1)	11/101	11/11	1.4 (0.7)
Other [a, b]	23	19	2.1 (1.4)	28	22	2.5 (2.9)

Notation: SD: standard deviation; n.a.: non applicable.

[a]: Other specialists reported in the Cycle + Usual physiotherapy group (more than one specialist could be reported by patients): allergy specialist (n=1), endocrinologist (n=3), epilepsy specialist (n=1), haematologist (n=5), infectious disease specialist (n=2), internal medicine physician (n=3), liver transplant surgeon (n=1),oncologist (n=4), ophthalmologist (n=4), radiologist (n=1), rheumatologist (n=1), thoracic surgeon (n=1), and urologist (n=1).
[b]: Other specialists reported in the Usual physiotherapy group (more than one specialist could be reported by patients): endocrinologist (n=2), geriatrician (n=3), haematologist (n=3), infectious disease specialist (n=3), internal medicine physician (n=4), liver transplant surgeon (n=1), oncologists (n=6), ophthalmologists (n=2), pain specialist (n=1), plastic surgeon (n=1), rheumatologist (n=1), thoracic surgeon (n=3), thrombosis specialist (n=1), urologist (n=1) and wound specialist (n=1).

© 2025 Tarride J-E et al. *JAMA Network Open*.

eTable 10. 90-Day Follow-Up Questionnaire: Other Health Care Professionals (Nonimputed Data)

Other healthcare professionals	**Cycling + Usual physiotherapy** (100/102 patients responded whether or not they had seen another healthcare professional since hospital discharge)			**Usual physiotherapy** (101/103 patients responded whether or not they had seen another healthcare professional since hospital discharge)		
	Number of patients reporting a visit	Number of patients providing the number of visits	Mean (SD) number of visits among those reporting a visit	Number of patients reporting a visit	Number of patients providing the number of visits	Mean (SD) number of visits among those reporting a visit
Chiropractor	0/100	0/0		2/101	1/2	3.0
Dietician	8/100	8/8	1.3 (0.5)	7/101	4/7	1.5 (1.0)
Personal Support Worker	4/100	4/4	25.3 (23.8)	3/101	1/3	70.0
Meals on Wheels	2/100	0/2	n.a.	1/101	0/1	n.a.
Nurse practitioner	7/100	7/7	1.9 (1.9)	5/101	2/5	1.0 (0.0)
Occupational Therapist	9/100	8/9	2.4 (2.0)	9/101	6/9	3.0 (4.4)
Private Nurse	1/100	0/1		1/101	1/1	2.0
Psychologist	1/100	1/1	4.0 (4.0)	2/101	2/1	2.5 (2.1)
Physiotherapist	17/100	15/17	4.6 (5.9)	18/101	14/18	6.3 (2.7)
Respiratory therapist	2/100	2/2	1.0 (0.0)	5/101	3/5	2.7 (1.5)
Speech language Therapist	1/100	1/1	1.0	4/101	3/4	4.7 (6.4)
Social Worker	0/100	0/0	n.a.	6/101	4/6	2.5 (1.9)
Transportation Services	0/100	0/0	n.a.	1/101	0/1	n.a.
Visiting Nurse	19/100	16/19	7.5 (10.2)	14/101	12/14	10.0 (11.1)
Other [a,]	3/100	3/3	2.3 (2.5)	6/101	6/6	3.8 (5/2)

Notation: SD: standard deviation; n.a.: not applicable.

[a]: Other healthcare professionals reported in the Cycle + Usual physiotherapy group (more than one healthcare professional could be reported by patients include): emergency technician (n=1), pharmacist (n=1), and, plastic technician (n=1) and "unknown but help for exercise" (n=1). Other healthcare professionals reported in the Usual physiotherapy group include: acupuncturist (n=1), diabetes specialist (n=1), energy healer (n=1), kinesiologist (n=1), osteopath (n=2).

© 2025 Tarride J-E et al. *JAMA Network Open*.

eTable 11. 90-Day Follow-Up Questionnaire: Days Spent in Facilities (Nonimputed Data)

	**Cycling +Usual physiotherapy** (100/102 patients responded whether or not they had been admitted to a healthcare facility since hospital discharge)			**Usual physiotherapy** (101/103 patients responded whether or not they had been admitted to a healthcare facility since hospital discharge)		
	Number of patients reporting an admission	Number of patients providing the number of days admitted	Mean (SD) number of days among those reporting days admitted	Number of patients reporting an admission	Number of patients providing the number of days admitted	Mean (SD) number of days among those reporting days admitted
Long term care days	3/100	3/3	27.7 (17.0)	4/101	4/4	24.0 (8.8)
Retirement home days	1/100	1/1	90.0 (n.a.)	0/101	0/0	n.a.
Assisted living days	3/100	1/3	72.0 (n.a.)	0/101	0/0	n.a.
Chronic care facility days	1/100	1/1	39.0 (n.a)	1/101	0/0	n.a.
Inpatient rehabilitation days	13/100	11/13	19.9 (9.8)	13/101	11/13	18.9 (16.3)
Other: Outpatient psychiatric hospital days	0/100	n.a.	n.a.	1/101	1/1	48
Other: Drug rehabilitation facility days	0/100	n.a.	n.a.	1/101	1/1	90

Notation: SD: standard deviation; n.a.: not applicable.

© 2025 Tarride J-E et al. *JAMA Network Open*.

eTable 12. Admission to Other Health Care Facilities Based on Living Status Before Index Admission: Cycling Plus Usual Physiotherapy (Nonimputed Data)

Number of patients indicating they spent days in [a]		Living status before index admission (N=8)			
		Home (independent)	Home (with unpaid caregiver assistance)	Retirement home	Assisted living facility
Retirement home	1/100	0	0	1	0
Assisted living facilities	3/100	1	0	0	2
Long-term care facility	3/100	3	0	0	0
Chronic care facility	1/100	1	0	0	0

[a]: Number of patients getting assigned costs based on number of days reported and baseline living status: 1 assigned to assisted living facilities; 3 assigned to long-term care facilities; and 1 to chronic care facility.

© 2025 Tarride J-E et al. *JAMA Network Open*.

eTable 13. Admission to Other Health Care Facilities Based on Living Status Before Index Admission: Usual Physiotherapy (Nonimputed Data)

Number of patients indicating they spent days in [a]		Living status before index admission (N=7)			
		Home (independent)	Home (with unpaid caregiver assistance)	Retirement home	Assisted living facility
Retirement home	0/101	0	0	0	0
Assisted living facility	0/101	0	0	0	0
Long-term care facility	4/101	2	1	1	0
Chronic care facility	1/101	1	0	0	0
Other facilities	2/101	2	0	0	0

[a]: Number of patients getting assigned costs based on number of days reported and baseline living status: 2 assigned to long-term care facilities; 1 to chronic care facility and 2 assigned to others (i.e., outpatient psychiatric hospital; drug rehabilitation facility).

.

© 2025 Tarride J-E et al. *JAMA Network Open*.

eTable 14. Assistance From Others and Work Time Lost (Nonimputed Data)

	**Cycling +Usual physiotherapy** (101/102 patients responded about their employment status and whether or not they had received assistance from others since hospital discharge)			**Usual physiotherapy** (101/103 patients responded about their employment status and whether or not they had received assistance from others since hospital discharge)		
	Number of patients reporting assistance from others or time off work	Number of patients with full data on number of hours	Mean (SD) number of hours	Number of patients reporting assistance from others or time off work	Number of patients with full data on number of hours	Mean (SD) number of hours
Assistance from others (hours)	39/101	22/39	98.8 (132.1)	48/101	31/48	126.9 (212.4)
Hours taken off work among working individuals providing assistance to patient	4/101	3/4	293.3 (433.4)	9/101	5/9	118.4 (74.9)
Patients taking time off work [a] (hours)	26/101	19/26	250.4 (200.6)	19/101	16/20	315.8 (145.0)

Notation: SD: standard deviation

[a]:*This represents the number of people that indicated that they were working full or part time prior to the hospitalization.

© 2025 Tarride J-E et al. *JAMA Network Open*.

eTable 15. Comparison of 90-Day Nonimputed and Imputed Health Care Resource Use

	**Cycling + Usual physiotherapy**		**Usual physiotherapy**	
	Nonimputed data Mean (SE)	Imputed data Mean (SE)	Nonimputed data Mean (SE)	Imputed data Mean (SE)
ICU days	0.38 (0.22)	0.36 (0.21)	0.10 (0.05)	0.10 (0.05)
Non-ICU days	1.61 (0.47)	1.47 (0.42)	0.89 (0.39)	0.83 (0.36)
ED visits	0.29 (0.06)	0.27 (0.06)	0.24 (0.05)	0.22 (0.04)
Family doctor	0.59 (0.10)	0.57 (0.09)	0.63 (0.09)	0.60 (0.09)
Specialist visits	0.90 (0.12)	0.85 (0.11)	0.79 (0.15)	0.90 (0.13)
Other health care professionals	2.40 (0.68)	2.15 (0.54)	2.70 (0.99)	2.18 (0.72)
Long term care days	0.60 (0.39)	0.52 (0.32)	0.71 (0.37)	0.65 (0.33)
Assisted living days	0.52 (0.52)	0.45 (0.44)	0.00 (0.00)	0.00 (0.00)
Chronic care facilities days	0.28 (0.28)	0.24 (0.24)	0.29 (0.29)	0.27 (0.27)
Inpatient rehabilitation days	1.88 (0.55)	1.73 (0.50)	1.81 (0.61)	1.57 (0.51)
Other Locations	0.00 (0.00)	0.00 (0.00)	0.95 (0.70)	0.95 (0.70)
Hours of assistance needed by others	27.09 (5.86)	24.63 (5.06)	36.07 (9.30)	32.94 (7.84)
Hours taken off work for those assisting patient	8.15 (5.87)	6.69 (4.77)	7.40 (2.61)	6.72 (2.33)
Hours taken off work by patient	39.25 (9.34)	37.19 (8.72)	42.93 (9.96)	40.35 (9.23)

Notations: ICU: intensive care unit; LOS: length of stay; SD: standard deviation; ED: emergency department.

© 2025 Tarride J-E et al. *JAMA Network Open*.

eTable 16. Comparison of Nonimputed Case and Imputed Health Care Resource Use: Specialist Visits

	Cycling + Usual physiotherapy		Usual physiotherapy	
	Nonimputed data Mean (SE) visits	Imputed data Mean (SE) visits	Nonimputed data Mean (SE) visits	Imputed data Mean (SE) visits
Cardiologist	0.08 (0.03)	0.09 (0.03)	0.11 (0.03)	0.10 (0.03)
Dentist	0.03 (0.02)	0.03 (0.01)	0.01 (0.01)	0.01 (0.01)
Dermatologist	0.00 (0.00)	0.00 (0.00)	0.01(0.01)	0.01 (0.01)
Ear Nose Throat	0.03 (0.02)	0.03 (0.02)	0.02 (0.02)	0.02 (0.02)
Gastroenterologist	0.06 (0.02)	0.06 (0.02)	0.04 (0.02)	0.04 (0.02)
Nephrologist	0.06 (0.02)	0.07 (0.02)	0.03 (0.02)	0.02 (0.01)
Neurologist	0.03 (0.02)	0.03 (0.02)	0.01(0.01)	0.01 (0.01)
Psychiatrist	0.00 (0.00)	0.00 (0.00)	0.01 (0.01)	0.02 (0.01)
Physiatrist	0.01 (0.01)	0.01 (0.01)	0.00 (0.00)	0.00 (0.00)
Respirologist	0.14 (0.04)	0.14 (0.04)	0.12 (0.03)	0.12 (0.03)
Surgeon	0.09 (0.04)	0.08 (0.03)	0.10 (0.03)	0.10 (0.03)
Other	0.34 (0.08)	0.32 (0.07)	0.47 (0.12)	0.43 (0.11)

Notation: SD: standard deviation.

© 2025 Tarride J-E et al. *JAMA Network Open*.

eTable 17. Comparison of Nonimputed and Imputed Health Care Resource Use: Other Health Care Professional Visits

	**Cycling + Usual physiotherapy**		**Usual physiotherapy**	
	Nonimputed data Mean (SE) visits	Imputed data Mean (SE) visits	Nonimputed data Mean (SE) visits	Imputed data Mean (SE) visits
Chiropractor	0.00 (0.00)	0.00 (0.00)	0.02 (0.02)	0.02 (0.02)
Dietician	0.07 (0.03)	0.07 (0.02)	0.04 (0.02)	0.04 (0.02)
Personal Support Worker	0.70 (0.45)	0.58 (0.37)	0.50 (0.50)	0.38 (0.38)
Nurse Practitioner	0.09 (0.05)	0.08 (0.04)	0.01 (0.01)	0.01 (0.01)
Occupational Therapist	0.13 (0.06)	0.12 (0.05)	0.13 (0.09)	0.10 (0.07)
Private Nurse	0.00 (0.00)	0.00 (0.00)	0.01 (0.01)	0.01 (0.01)
Psychologist	0.03 (0.03)	0.02 (0.02)	0.03 (0.03)	0.03 (0.02)
Physiotherapist	0.49 (0.20)	0.43 (0.16)	0.63 (0.26)	0.55 (0.21)
Respiratory Therapist	0.01 (0.01)	0.01 (0.01)	0.06 (0.04)	0.06 (0.04)
Speech Language Therapist	0.01 (0.01)	0.01 (0.01)	0.10 (0.08)	0.08 (0.07)
Social Worker	0.00 (0.00)	0.00 (0.00)	0.07 (0.04)	0.06 (0.03)
Transportation Services	0.00 (0.00)	0.00 (0.00)	0.00 (0.00)	0.00 (0.00)
Visiting Nurse	0.85 (0.35)	0.78 (0.30)	0.85 (0.35)	0.71 (0.28)
Other	0.06 (0.04)	0.05 (0.04)	0.14 (0.09)	0.13 (0.09)

Notation: SD: standard deviation.

© 2025 Tarride J-E et al. *JAMA Network Open*.

eTable 18. Comparison of Nonimputed and Imputed 90-Day Follow-Up Costs and 90-Day EQ-5D-5L

	Cycling + Usual Physiotherapy		Usual physiotherapy	
	**Mean (SE) Nonimputed costs**	**Mean (SE) Imputed costs**	**Mean (SE) Nonimputed costs**	**Mean (SE) Imputed costs**
ICU days	$1,422 ($821)	$1,338 ($768)	$389 ($204)	$363 ($182)
Non-ICU days	$1,888 ($550)	$1,724 ($489)	$1043 ($461)	$975 ($419)
ED visits	$107 ($23)	$99 ($20)	$86 ($17)	$78 ($15)
Family doctor	$23 ($4)	$22 ($3)	$24 ($4)	$23 ($3)
Specialist visits	$140 ($18)	$133 ($17)	$141 ($21)	$133 ($19)
Other health care professionals	$57 ($15)	$50 ($12)	$66 ($21)	$54 ($15)
Long term care days	$174 ($112)	$150 ($92)	$204 ($106)	$355 ($293)
Assisted living days	$64 ($64)	$55 ($55)	$0 ($0)	$0 ($0)
Chronic care facilities days	$355 ($355)	$287 ($284)	$340 ($340)	$319 ($319)
Inpatient rehabilitation days	$2101 ($611)	$1,938 ($557)	$2,024 ($685)	$1,760 ($570)
Other locations	$0 ($0)	$0 ($0)	$341 ($280)	$355 ($296)
Hours of assistance needed	$556 ($120)	$506 ($104)	$741 ($191)	$671 ($159)
Hour taken off work for those assisting patients	$377 ($272)	$310 ($221)	$342 ($121)	$312 ($110)
Hours of work taken off by patient	$1,817 ($432)	$1,721($404)	$1987 ($461)	$1,867 ($427)
**90 day EQ 5D utility** [a]	0.8013 (0.0166)	0.7975 (0.0151)	0.7872 (0.0193)	0.7754 (0.0184)

Notations: ICU: intensive care unit; ED: emergency department; SE: standard error.

[a]: EQ-5D utilities represent patient health states with 1 representing full health and 0 representing death.

© 2025 Tarride J-E et al. *JAMA Network Open*.

eReferences

1. Government of Ontario. Schedule of Benefits for Physician Services. Accessed October 20, 2024, https://www.ontario.ca/files/2024-08/moh-schedule-benefit-2024-08-30.pdf
2. Kho ME, Reid J, Molloy AJ, et al. Critical Care Cycling to Improve Lower Extremity Strength (CYCLE): protocol for an international, multicentre randomised clinical trial of early in-bed cycling for mechanically ventilated patients. *BMJ Open*. Jun 23 2023;13(6):e075685. doi:10.1136/bmjopen-2023-075685
3. Government of Canada. Job Bank. Hourly wages for Ontario. Accessed October 20, 2024, https://www.jobbank.gc.ca/trend-analysis/search-wages?wbdisable=true
4. Canadian Institute for Health Information. Hospital spending: Focus on the emergency department. Ottawa, ON: CIHI; 2020. Accessed October 20, 2024, https://www.cihi.ca/sites/default/files/document/hospital-spending-highlights-2020-en.pdf
5. Government of Canada. Canada Mortgage and Housing Corporation (CMHC). 2021 Seniors Housing Survey: Learn more from our insights. https://www.cmhc-schl.gc.ca/blog/2021/2021-seniors-housing-survey-learn-more-insights
6. Governement of Ontario. Long-term care homes level-of-care per diem funding summary. Accessed October 21, 2024, https://www.ontario.ca/page/long-term-care-home-level-care-diem-funding-summary
7. Government of Ontario. Hospital Chronic Care Co-Payment. Accessed Octobe 21, 2024, https://www.ontario.ca/page/hospital-chronic-care-co-payment#section-1Hospital%20Chronic%20Care%20Co-Payment%20|%20ontario.ca%22
8. Addiction Rehab Toronto. How Much Does Rehab Cost in Ontario? Accessed November 22, 2024, https://addictionrehabtoronto.ca/how-much-does-rehab-cost-in-ontario/
9. Health Quality Ontario. Internet-Delivered Cognitive Behavioural Therapy for Major Depression and Anxiety Disorders: A Health Technology Assessment. *Ont Health Technol Assess Ser*. 2019;19(6):1-199.
10. Statistics Canada. Employment, average hourly and weekly earnings (including overtime), and average weekly hours for the industrial aggregate excluding unclassified businesses, monthly, seasonally adjusted. Accessed October 20, 2024, https://www150.statcan.gc.ca/t1/tbl1/en/tv.action?pid=1410022201
11. Willow Dental. Costs You Can Expect For Your Dental Procedure And Insurance Coverage Options. Accessed November 22, 2024, https://willowdental.com/costs-can-expect-dental-procedure-insurance-coverage-options/
12. Ontario Chiropractor Association (OCA). OCA Recommended Service Codes and Fee Schedule. Accessed October 21, 2024, https://chiropractic.on.ca/wp-content/uploads/2022-Fee-Schedule-FINAL-1-Dec-2022.pdf
13. Meals on Wheels. Frequently Asked Questions. Accessed October 21, 2024, https://www.mealsonwheels-ottawa.org/about-us/faq/

© 2025 Tarride J-E et al. *JAMA Network Open*.

14. Bay Observer. Opinion: Is DARTS The Only Way To Travel? Accessed October 21, 2024, https://bayobserver.ca/opinion-is-darts-the-only-way-to-travel/#google_vignette

© 2025 Tarride J-E et al. *JAMA Network Open*.
