# Supplementary material for: Cost-Effectiveness of In-Bed Cycling and Routine Physiotherapy for Patients Receiving Mechanical Ventilation
Source: JAMA Netw Open. 2025 Sep 8;8(9):e2529399. doi: 10.1001/jamanetworkopen.2025.29399 (PMC12418132; doi:10.1001/jamanetworkopen.2025.29399)
Supplement: Supplement 2. — Group Information [file jamanetwopen-e2529399-s002.pdf]

Supplemental Online Content: Nonauthor Collaborators
*First name, last name, and suffix (if applicable) are required and will appear in PubMed.

***Group Name(s): Canadian Critical Care Trials Group**

***First Name and Middle Initial(s)**	***Last Name**	***Suffix (eg, Jr, III)**	Academic Degrees	Institution	Location (city, state/province, country)	Role or Contribution, eg, chair, principal investigator	Group (if more than 1 Group listed in the byline) and/or Subgroup (eg, Steering Committee)
Kristen	Abercombie		MPT	London Health Sciences Centre	London, Ontario, Canada	Interventionist	
Daana	Ajami		M.Sc P.T.	St Joseph's Healthcare Hamilton	Hamilton, Ontario, Canada	Interventionist	
Mimi	Anderson		B.Sc PT	Kingston General Hospital	Kingston, Ontario, Canada	Interventionist	
Daniel	Aranda		B.Sc PT	Niagara Health St Catherines	St Catherines, Ontario, Canada	Interventionist	
Sabrina	Araujo de Franca		BaSN	Hôpital du Sacré-Coeur de Montréal	Montréal, Quebec, Canada	Research Assistant	
Cortney	Armstrong		BS, PT	Juravinski Hospital	Hamilton, Ontario, Canada	Interventionist	Blind Outcome Assessor
Huy-Thanh	Bach		M.Sc PT	Hôpital du Sacré-Coeur de Montréal	Montréal, Quebec, Canada	Blinded Outcome Assessor	
Zoe	Bacolas		B.Bioscience, B.Sci, DPT	Austin Hospital	Victoria, Australia	Blinded Outcome Assessor	Interventionist
William	Baron			Hôpital Hôtel-Dieu De Lévis	Lévis, Quebec, Canada	Research Assistant	
Andrea	Barrass		B.Sc PT	Ottawa Civic Hospital	Ottawa, Ontario, Canada	Blinded Outcome Assessor	
Kristen	Baxter		M.Sc PT	Mount Sinai Hospital	Toronto, Ontario, Canada	Interventionist	
Genevieve	Beatuchamp-Vien		M.Sc PT	Hôpital du Sacré-Coeur de Montréal	Montréal, Quebec, Canada	Blinded Outcome Assessor	Interventionist
Amanda	Beaty		DPT	Duke University Hospital	Durham, North Carolina, USA	Research Assistant	
Rosalie	Beaudoin			Hôpital Hôtel-Dieu De Lévis	Lévis, Quebec, Canada	Research Assistant	
Pier-Olivier	Beaumont			Hôpital Hôtel-Dieu De Lévis	Lévis, Quebec, Canada	Research Assistant	

Supplemental Online Content: Nonauthor Collaborators
*First name, last name, and suffix (if applicable) are required and will appear in PubMed.

***First Name and Middle Initial(s)**	***Last Name**	***Suffix (eg, Jr, III)**	Academic Degrees	Institution	Location (city, state/province, country)	Role or Contribution, eg, chair, principal investigator	Group (if more than 1 Group listed in the byline) and/or Subgroup (eg, Steering Committee)
Julia	Becevel		B.Sc, MBA, MPH	Niagara Health St Catherines	St Catherines, Ontario, Canada	Student	
Andréane	Bédard			Hôpital Hôtel-Dieu De Lévis	Lévis, Quebec, Canada	Research Assistant	
Anne-Julie	Belanger			Hôpital Hôtel-Dieu De Lévis	Lévis, Quebec, Canada	Research Assistant	
Julie	Bélisle		RN	Hôpital Fleurimont – Sherbrooke (CHUS)	Sherbrooke, Quebec, Cana	Research Assistant	
Emilie	Belley-Cote		M.D., Ph.D	Hamilton General Hospital	Hamilton, Ontario, Canada	Site Co-Investigator	
Vickie	Bergeron		M.Sc PT	Hôpital du Sacré-Coeur de Montréal	Montréal, Quebec, Canada	Interventionist	
Francis	Bernard		MD	Hôpital du Sacré-Coeur de Montréal	Montréal, Quebec, Canada	Co-Investigator	
Andréanne	Bernatchez			Hôpital Hôtel-Dieu De Lévis	Lévis, Quebec, Canada	Research Assistant	
Xavier	Bernet		M.Sc PT	Hôpital du Sacré-Coeur de Montréal	Montréal, Quebec, Canada	Interventionist	Blinded Outcome Assessor
Jennifer	Bessette		M.Sc PT	Hôpital du Sacré-Coeur de Montréal	Montréal, Quebec, Canada	Interventionist	Blinded Outcome Assessor
Stephanie	Biffaro		B.Biomed Sc, DPT	Austin Hospital	Victoria, Australia	Blinded Outcome Assessor	
Elizabeth	Bitzer		BH.Sc PT	Niagara Health St Catherines	St Catherines, Ontario, Canada	Blinded Outcome Assessor	
Grant	Bitzer		DPT, EdD	Duke University Hospital	Durham, North Carolina, USA	Interventionist	Research Assistant
Karen	Bolduc		B.Sc PT	Hôpital Hôtel-Dieu De Lévis	Lévis, Quebec, Canada	Interventionist	
Simon	Bordeleau		MD, FRCPC	Hôpital Hôtel-Dieu De Lévis	Lévis, Quebec, Canada	Co-Investigator	
Marie-Pier	Bouchard			Hôpital Fleurimont – Sherbrooke (CHUS)	Sherbrooke, Quebec, Cana	Research Assistant	
Katherine	Boucher			Hôpital Hôtel-Dieu De Lévis	Lévis, Quebec, Canada	Research Assistant	
Kristina	Boucher			Hôpital Hôtel-Dieu De Lévis	Lévis, Quebec, Canada	Research Assistant	

Supplemental Online Content: Nonauthor Collaborators
*First name, last name, and suffix (if applicable) are required and will appear in PubMed.

*First Name and Middle Initial(s)	*Last Name	*Suffix (eg, Jr, III)	Academic Degrees	Institution	Location (city, state/province, country)	Role or Contribution, eg, chair, principal investigator	Group (if more than 1 Group listed in the byline) and/or Subgroup (eg, Steering Committee)
Arianne	Boudreault			Hôpital Hôtel-Dieu De Lévis	Lévis, Quebec, Canada	Research Assistant	
Mireille	Boutin			Hôpital Hôtel-Dieu De Lévis	Lévis, Quebec, Canada	Research Assistant	
Gordon	Boyd		MD, PhD, I	Kingston General Hospital	Kingston, Ontario, Canada	Site Co-Investigator	
Tracy	Boyd		M.Sc	Kingston General Hospital	Kingston, Ontario, Canada	Research Coordinator	
Nick	Bradley			Mount Sinai Hospital	Toronto, Ontario, Canada		
Alaina	Brescia		DPT	University of Maryland	Baltimore, Maryland, USA	Blinded Outcome Assessor	
Ashley	Bridgen		M.Sc PT	Kingston General Hospital	Kingston, Ontario, Canada	Blinded Outcome Assessor	
Tania	Brittain		B.Sc PT	Juravinski Hospital	Hamilton, Ontario, Canada	Interventionist	Blind Outcome Assessor
Sarah	Brown		M.Sc PT	St. Michael's Hospital	Toronto, Ontario, Canada	Interventionist	
Stephanie	Bryan		B.Sc PT	Kingston General Hospital	Kingston, Ontario, Canada	Blinded Outcome Assessor	
Nicole	Burgess		B.Biotech and Medical Research, DPT	Austin Hospital	Victoria, Australia	Interventionist	Research Assistant
Mercedes	Camargo-Penuela			Niagara Health St Catherines	St Catherines, Ontario, Canada		
Eileen	Campbell		CCRP	London Health Sciences Centre	London, Ontario, Canada	Research Coordinator	
Laura	Camposilvan		B.Sc Kin, OTA/PTA	St Joseph's Healthcare Hamilton	Hamilton, Ontario, Canada	Interventionist	Blind Outcome Assessor
Elaine	Carbonneau		RN	Hôpital Fleurimont – Sherbrooke (CHUS)	Sherbrooke, Quebec, Cana	Research Coordinator	

Supplemental Online Content: Nonauthor Collaborators
*First name, last name, and suffix (if applicable) are required and will appear in PubMed.

***First Name and Middle Initial(s)**	***Last Name**	***Suffix (eg, Jr, III)**	Academic Degrees	Institution	Location (city, state/province, country)	Role or Contribution, eg, chair, principal investigator	Group (if more than 1 Group listed in the byline) and/or Subgroup (eg, Steering Committee)
Christina	Carile		DPT	Niagara Health St Catherines	St Catherines, Ontario, Canada	Interventionist	
Eve-Marie	Casavant			Hôpital Fleurimont – Sherbrooke (CHUS)	Sherbrooke, Quebec, Cana	Research Assistant	
Alexandros	Cavayas		MD	Hôpital du Sacré-Coeur de Montréal	Montréal, Quebec, Canada	Co-Investigator	
Christine	Cavers		B.Sc PT	Niagara Health St Catherines	St Catherines, Ontario, Canada	Research Assistant	
Erik	Celikovic			Hôpital Hôtel-Dieu De Lévis	Lévis, Quebec, Canada	Research Assistant	
Diana	Challis		B.Sc PT	Kingston General Hospital	Kingston, Ontario, Canada	Blinded Outcome Assessor	
Josianne	Charbonneau		M.Sc PT	Hôpital du Sacré-Coeur de Montréal	Montréal, Quebec, Canada	Interventionist	Blinded Outcome Assessor
Emmanuel	Charbonney		MD, PhD	Hôpital du Sacré-Coeur de Montréal	Montréal, Quebec, Canada	Co-Investigator	
Mitali	Chopra		H.B.Sc	St Joseph's Healthcare Hamilton	Hamilton, Ontario, Canada	Methods Centre	
Selina	Chow		M.D.	St Joseph's Healthcare Hamilton	Hamilton, Ontario, Canada	Student	
France	Clarke		RRT	St Joseph's Healthcare Hamilton	Hamilton, Ontario, Canada	Research Coordinator	
Robert	Coke		M.B.B.S, FRCPC	Brantford General Hospital	Brantford, Ontario, Canada	Site Co-Investigator	
Eileen	Connor		DPT	University of Maryland	Baltimore, Maryland, USA	Blinded Outcome Assessor	
Natalie	Constantin		M.Sc P.T.	St Joseph's Healthcare Hamilton	Hamilton, Ontario, Canada	Student	
Kelly	Cooke		OTA/PTA	Brantford General Hospital	Brantford, Ontario, Canada	Blinded Outcome Assessor	
Caity	Cooper		BSc, OTA/PTA	St. Michael's Hospital	Toronto, Ontario, Canada	Interventionist	

Supplemental Online Content: Nonauthor Collaborators
*First name, last name, and suffix (if applicable) are required and will appear in PubMed.

*First Name and Middle Initial(s)	*Last Name	*Suffix (eg, Jr, III)	Academic Degrees	Institution	Location (city, state/province, country)	Role or Contribution, eg, chair, principal investigator	Group (if more than 1 Group listed in the byline) and/or Subgroup (eg, Steering Committee)
Mary	Copland		B.A	Brantford General Hospital	Brantford, Ontario, Canada	Research Assistant	
Melanie	Cosntantin			Hôpital Hôtel-Dieu De Lévis	Lévis, Quebec, Canada	ICU Nurse	
Emile	Côté			Hôpital Hôtel-Dieu De Lévis	Lévis, Quebec, Canada	Research Coordinator	
Line	Côté		RN	Hôpital Fleurimont – Sherbrooke (CHUS)	Sherbrooke, Quebec, Cana	Research Coordinator	
Laurie	Couture			Hôpital Hôtel-Dieu De Lévis	Lévis, Quebec, Canada	Research Assistant	
Vanessa	Couture			Hôpital Hôtel-Dieu De Lévis	Lévis, Quebec, Canada	Research Assistant	
Michelle	Cummings		B.Sc PT	Ottawa Civic Hospital	Ottawa, Ontario, Canada	Interventionist	Blinded Outcome Assessor
Jennifer	Curry		MPT	London Health Sciences Centre	London, Ontario, Canada	Interventionist	
Rohun	Dalal		BS	University of Maryland	Baltimore, Maryland, USA	Research Assistant	
Anne	Davenport		DPT	Duke University Hospital	Durham, North Carolina, USA	Research Assistant	
Shivaun	Davidson		M.Sc, PT	Juravinski Hospital	Hamilton, Ontario, Canada	Interventionist	Blind Outcome Assessor
William	Dechert		M.Sc	Brantford General Hospital	Brantford, Ontario, Canada	Research Coordinator	
Maura	Deeley		CCRP	University of Maryland	Baltimore, Maryland, USA	Research Coordinator	
Marianne	Denommee		M.Sc PT	Hôpital du Sacré-Coeur de Montréal	Montréal, Quebec, Canada	Blinded Outcome Assessor	
Shelley	DeRooy		DPT	Ottawa General Hospital	Ottawa, Ontario, Canada	Blinded Outcome Assessor	
Caitlin	Devanny		B.Physio	Austin Hospital	Victoria, Australia	Blinded Outcome Assessor	
Carly	Di Diodato		RN B.ScN	Juravinski Hospital	Hamilton, Ontario, Canada	Research Coordinator	

Supplemental Online Content: Nonauthor Collaborators
*First name, last name, and suffix (if applicable) are required and will appear in PubMed.

***First Name and Middle Initial(s)**	***Last Name**	***Suffix (eg, Jr, III)**	Academic Degrees	Institution	Location (city, state/province, country)	Role or Contribution, eg, chair, principal investigator	Group (if more than 1 Group listed in the byline) and/or Subgroup (eg, Steering Committee)
Katie	DiAnthony		DPT	University of Maryland	Baltimore, Maryland, USA	Blinded Outcome Assessor	
Joanna C	Dionne		M.D., Ph.D	Juravinski Hospital	Hamilton, Ontario, Canada	Site Co-Investigator	
Grace	Ditzenberger		DPT	Duke University Hospital	Durham, North Carolina, USA	Research Assistant	Other
Ashleigh	Donavon		B.Physio	Austin Hospital	Victoria, Australia	Research Coordinator	Blinded Outcome Assessor
Sara	Dorn		DPT	Duke University Hospital	Durham, North Carolina, USA	Interventionist	
Claudia	Dos Santos		MD	St. Michael's Hospital	Toronto, Ontario, Canada	Co-Investigator	
Christine	Drouin		MD	Hôpital Hôtel-Dieu De Lévis	Lévis, Quebec, Canada	Co-Investigator	
John	Drover		MD, FRCS	Kingston General Hospital	Kingston, Ontario, Canada	Site Co-Investigator	
Angela	Dueck		B.Sc PT	Niagara Health St Catherines	St Catherines, Ontario, Canada	Interventionist	
Benoit	Duhaime		MD	Hôpital Hôtel-Dieu De Lévis	Lévis, Quebec, Canada	Co-Investigator	
Jennifer	Duley		M.Sc P.T.	Hamilton General Hospital	Hamilton, Ontario, Canada	Blind Outcome Assessor	
Danielle	Dunlop		B.Physio	Austin Hospital	Victoria, Australia	Interventionist	Blinded Outcome Assessor
Suzanna	Dunn		DPT	University of Maryland	Baltimore, Maryland, USA	Blinded Outcome Assessor	
Mathilde	Duplaix		M.Sc	Hôpital du Sacré-Coeur de Montréal	Montréal, Quebec, Canada	Research Assistant	
Estel	Duquet-Deblois			Hôpital Hôtel-Dieu De Lévis	Lévis, Quebec, Canada	Research Coordinator	
Aarthy	Ehaparaseelan			Niagara Health St Catherines	St Catherines, Ontario, Canada	Research Assistant	

Supplemental Online Content: Nonauthor Collaborators
*First name, last name, and suffix (if applicable) are required and will appear in PubMed.

***First Name and Middle Initial(s)**	***Last Name**	***Suffix (eg, Jr, III)**	Academic Degrees	Institution	Location (city, state/province, country)	Role or Contribution, eg, chair, principal investigator	Group (if more than 1 Group listed in the byline) and/or Subgroup (eg, Steering Committee)
Hussein	El Maoula		M.Sc PT	Hôpital du Sacré-Coeur de Montréal	Montréal, Quebec, Canada	Blinded Outcome Assessor	
Jamie	Emery		M.Sc PT	Ottawa Civic Hospital	Ottawa, Ontario, Canada	Blinded Outcome Assessor	
Jamie	Emery		M.Sc PT	Ottawa General Hospital	Ottawa, Ontario, Canada	Interventionist	Blinded Outcome Assessor
Christopher	Farley		M.Sc PT, I	Brantford General Hospital; Juravinski Hospital	Brantford, Ontario, Canada ; Hamilton, Ontario, Canada	Blinded Outcome Assessor	Interventionist
Deanna	Feltracco		M.Sc PT	St. Michael's Hospital	Toronto, Ontario, Canada	Interventionist	
Debbie	Fenwick		(Hons)BSc .PT, Ph.D	Niagara Health St Catherines	St Catherines, Ontario, Canada	Interventionist	
Eszter	Finta		B.Sc PT	Ottawa Civic Hospital	Ottawa, Ontario, Canada	Blinded Outcome Assessor	
Tanya	Forward		B.Sc PT	Ottawa Civic Hospital	Ottawa, Ontario, Canada	Blinded Outcome Assessor	
Julia	Fournier		M.Sc	Kingston General Hospital	Kingston, Ontario, Canada	Administration	
Jan	Friedrich		MD, PhD	St. Michael's Hospital	Toronto, Ontario, Canada	Co-Investigator	
Michelle	Fung		B.Sc OT , MSc	St Joseph's Healthcare Hamilton	Hamilton, Ontario, Canada	Methods Centre	
Siobhan	Gallagher		B.Sc PT	St. Michael's Hospital	Toronto, Ontario, Canada	Interventionist	
Wendy	Galloway		M.Sc P.T.	St Joseph's Healthcare Hamilton	Hamilton, Ontario, Canada	Blinded Outcome Assessor	
Nikita	Gandhi		M.Sc PT	St. Michael's Hospital	Toronto, Ontario, Canada	Interventionist	

Supplemental Online Content: Nonauthor Collaborators
*First name, last name, and suffix (if applicable) are required and will appear in PubMed.

***First Name and Middle Initial(s)**	***Last Name**	***Suffix (eg, Jr, III)**	Academic Degrees	Institution	Location (city, state/province, country)	Role or Contribution, eg, chair, principal investigator	Group (if more than 1 Group listed in the byline) and/or Subgroup (eg, Steering Committee)
Sarah	Garant Gignac		M.Sc PT	Hôpital du Sacré-Coeur de Montréal	Montréal, Quebec, Canada	Blinded Outcome Assessor	
Frances	Garner		B.Sc PT	St Joseph's Healthcare Hamilton	Hamilton, Ontario, Canada	Blinded Outcome Assessor	
Marie-Carmel	Gédéon		B.Sc PT	Hôpital du Sacré-Coeur de Montréal	Montréal, Quebec, Canada	Blinded Outcome Assessor	
Jessika	Giguere		B.Sc PT	Hôpital Hôtel-Dieu De Lévis	Lévis, Quebec, Canada	Interventionist	
Pascale	Girard		MScAPT	Hôpital Fleurimont – Sherbrooke (CHUS)	Sherbrooke, Quebec, Cana	Interventionist	Blinded Outcome Assessor
Brigette	Gomes		BSN	Ottawa Civic Hospital	Ottawa, Ontario, Canada	Research Coordinator	
Karla	Gorman		M.Sc PT	Brantford General Hospital	Brantford, Ontario, Canada	Blinded Outcome Assessor	
Kylie	Graham		B.Physio	Austin Hospital	Victoria, Australia	Blinded Outcome Assessor	
Karlyn S	Green		DPT	Duke University Hospital	Durham, North Carolina, USA	Blinded Outcome Assessor	
Samantha H	Green		MSOT, OTR/L	Duke University Hospital	Durham, North Carolina, USA	Interventionist	
Stephanie	Green		B.Sc PT	Ottawa General Hospital	Ottawa, Ontario, Canada	Blinded Outcome Assessor	
Justine	Grégoire			Hôpital Fleurimont – Sherbrooke (CHUS)	Sherbrooke, Quebec, Cana	Research Coordinator	
Anna	Gregory		M.Sc PT	Ottawa Civic Hospital	Ottawa, Ontario, Canada	Interventionist	
Brian	Grondin-Beaudoin		MD	Hôpital Fleurimont – Sherbrooke (CHUS)	Sherbrooke, Quebec, Cana	Co-Investigator	
Stephanie	Guilbault		B.Sc PT	Hôpital Hôtel-Dieu De Lévis	Lévis, Quebec, Canada	Interventionist	
Chantal	Guillemette		RN	Hôpital Fleurimont – Sherbrooke (CHUS)	Sherbrooke, Quebec, Cana	Interventionist	

Supplemental Online Content: Nonauthor Collaborators
*First name, last name, and suffix (if applicable) are required and will appear in PubMed.

***First Name and Middle Initial(s)**	***Last Name**	***Suffix (eg, Jr, III)**	Academic Degrees	Institution	Location (city, state/province, country)	Role or Contribution, eg, chair, principal investigator	Group (if more than 1 Group listed in the byline) and/or Subgroup (eg, Steering Committee)
Jessica	Haines		BScN, RN	Ottawa General Hospital	Ottawa, Ontario, Canada	Research Coordinator	
Hassan	Haji		MD	University of Maryland	Baltimore, Maryland, USA	Research Assistant	
Claudia	Hamel		B.Sc PT	Hôpital Hôtel-Dieu De Lévis	Lévis, Quebec, Canada	Interventionist	
Yannick	Hamelin			Hôpital Hôtel-Dieu De Lévis	Lévis, Quebec, Canada	Research Assistant	
Michaela	Hanley		M.Sc	Kingston General Hospital	Kingston, Ontario, Canada	Research Coordinator	
Quincy	Hanna		B.A	St Joseph's Healthcare Hamilton	Hamilton, Ontario, Canada	Student	
Thelma	Harrington		RRT	University of Maryland	Baltimore, Maryland, USA	Research Coordinator	
Hibaa	Hasan		H.B.Sc	St Joseph's Healthcare Hamilton	Hamilton, Ontario, Canada	Student	
Jacqueline	Hockridge		B.Sc PT	Hamilton General Hospital	Hamilton, Ontario, Canada	Blind Outcome Assessor	
Jennifer	Hodder		BScN	St. Michael's Hospital	Toronto, Ontario, Canada	Research Coordinator	
Jen	Hoogenes		PhD, MPH, MSc, MS	St Joseph's Healthcare Hamilton	Hamilton, Ontario, Canada	Methods Centre	
Diana	Horobetz		M.Sc PT	St. Michael's Hospital	Toronto, Ontario, Canada	Blinded Outcome Assessor	
Mary	Hoyt		DPT	University of Maryland	Baltimore, Maryland, USA	Interventionist	
Miranda	Hunt		B.A	Kingston General Hospital	Kingston, Ontario, Canada	Research Coordinator	
Abby	Hurd		M.Sc P.T.	St Joseph's Healthcare Hamilton	Hamilton, Ontario, Canada	Methods Centre	Interventionist
Michelle	Iorio		DPT	Duke University Hospital	Durham, North Carolina, USA	Interventionist	

Supplemental Online Content: Nonauthor Collaborators
*First name, last name, and suffix (if applicable) are required and will appear in PubMed.

***First Name and Middle Initial(s)**	***Last Name**	***Suffix (eg, Jr, III)**	Academic Degrees	Institution	Location (city, state/province, country)	Role or Contribution, eg, chair, principal investigator	Group (if more than 1 Group listed in the byline) and/or Subgroup (eg, Steering Committee)
Michele	Isenor		B.Sc PT	London Health Sciences Centre	London, Ontario, Canada	Interventionist	
Ivone	Ivone		M.Sc P.T.	Hamilton General Hospital	Hamilton, Ontario, Canada	Interventionist	Blind Outcome Assessor
Steph	Jameson		B.Physio	Austin Hospital	Victoria, Australia	Research Coordinator	Interventionist
Rose	Johnston		B.Sc PT	Ottawa Civic Hospital	Ottawa, Ontario, Canada	Interventionist	
Jane	Jomy			Niagara Health St Catherines	St Catherines, Ontario, Canada	Research Assistant	
Veronique	Julien			Hôpital Hôtel-Dieu De Lévis	Lévis, Quebec, Canada	Research Assistant	
Annick	Jutras			Hôpital Hôtel-Dieu De Lévis	Lévis, Quebec, Canada	Research Coordinator	
Jennifer	Kaisen		BS	Duke University Hospital	Durham, North Carolina, USA	Research Coordinator	
Melissa	Kandel		MSOT	Duke University Hospital	Durham, North Carolina, USA	Blinded Outcome Assessor	
Hassan	Kashif		CCRA , M.[illegible]	Hamilton General Hospital	Hamilton, Ontario, Canada	Research Coordinator	
Anne	Kelly		OTR/L	Duke University Hospital	Durham, North Carolina, USA	Blinded Outcome Assessor	
Alia	Khaled		B.H.Sc, MSc	Juravinski Hospital	Hamilton, Ontario, Canada	Data Entry	
Imrana	Khalid		MD	St. Michael's Hospital	Toronto, Ontario, Canada	Research Assistant	
Tamara	Klintworth-Kirk		DPT	Duke University Hospital	Durham, North Carolina, USA	Blinded Outcome Assessor	
Brett	Koermer		DPT	Duke University Hospital	Durham, North Carolina, USA	Research Coordinator	Interventionist
Brett	Koermer		DPT	Duke University Hospital	Durham, North Carolina, USA	Research Coordinator	

Supplemental Online Content: Nonauthor Collaborators
*First name, last name, and suffix (if applicable) are required and will appear in PubMed.

***First Name and Middle Initial(s)**	***Last Name**	***Suffix (eg, Jr, III)**	Academic Degrees	Institution	Location (city, state/province, country)	Role or Contribution, eg, chair, principal investigator	Group (if more than 1 Group listed in the byline) and/or Subgroup (eg, Steering Committee)
Olga	Kolesnik		MD	University of Maryland	Baltimore, Maryland, USA	Research Coordinator	
Estelle	Kyriacou		B.Health Sci, M.Physio	Austin Hospital	Victoria, Australia	Blinded Outcome Assessor	
Ann	Laberge		MD	Hôpital Hôtel-Dieu De Lévis	Lévis, Quebec, Canada	Co-Investigator	
Olivier	Lachance		MD	Hôpital Hôtel-Dieu De Lévis	Lévis, Quebec, Canada	Co-Investigator	
Philippe	Lachance		MD	Hôpital Hôtel-Dieu De Lévis	Lévis, Quebec, Canada	Co-Investigator	
Jeanne	Lacombe		M.Sc PT	Hôpital du Sacré-Coeur de Montréal	Montréal, Quebec, Canada	Blinded Outcome Assessor	
Jeff	Lacouvee		MScAPT	Hôpital Fleurimont – Sherbrooke (CHUS)	Sherbrooke, Quebec, Cana	Interventionist	
Marilene	Ladouceur			Hôpital Fleurimont – Sherbrooke (CHUS)	Sherbrooke, Quebec, Cana	Research Coordinator	
Christine	Lafond		MScAPT	Hôpital Fleurimont – Sherbrooke (CHUS)	Sherbrooke, Quebec, Cana	Interventionist	Blinded Outcome Assessor
Julia	Lainer Palacos		M.Sc	Hôpital du Sacré-Coeur de Montréal	Montréal, Quebec, Canada	Research Assistant	
Francois	Lamontagne		MD	Hôpital Fleurimont – Sherbrooke (CHUS)	Sherbrooke, Quebec, Cana	Co-Investigator	
Josee	Lamontagne		B.Sc PT	Ottawa General Hospital	Ottawa, Ontario, Canada	Interventionist	
Lysandre	Lamontagne-Montminy		M.Sc PT	Hôpital du Sacré-Coeur de Montréal	Montréal, Quebec, Canada	Interventionist	
Charles	Landoni			Brantford General Hospital	Brantford, Ontario, Canada	Interventionist	
Amilie	Langlois		M.Sc PT	Hôpital du Sacré-Coeur de Montréal	Montréal, Quebec, Canada	Interventionist	
Heather	Langlois		B.Sc	Ottawa General Hospital	Ottawa, Ontario, Canada	Research Assistant	

Supplemental Online Content: Nonauthor Collaborators
*First name, last name, and suffix (if applicable) are required and will appear in PubMed.

***First Name and Middle Initial(s)**	***Last Name**	***Suffix (eg, Jr, III)**	Academic Degrees	Institution	Location (city, state/province, country)	Role or Contribution, eg, chair, principal investigator	Group (if more than 1 Group listed in the byline) and/or Subgroup (eg, Steering Committee)
Tania	Larsen		PT, Ph.D	London Health Sciences Centre	London, Ontario, Canada	Interventionist	
Lise	Lavoie			Hôpital Hôtel-Dieu De Lévis	Lévis, Quebec, Canada	Research Assistant	
Carole-Anne	Lavoie-Berard		MD	Hôpital Hôtel-Dieu De Lévis	Lévis, Quebec, Canada	Co-Investigator	
Julien	Le Beller			Hôpital Hôtel-Dieu De Lévis	Lévis, Quebec, Canada	Research Coordinator	
Sylvie	LeBlanc		B.Sc PT	Niagara Health St Catherines	St Catherines, Ontario, Canada	Interventionist	
Liane	LeClair		M.Sc	Ottawa Civic Hospital	Ottawa, Ontario, Canada	Research Assistant	
Marc-Andre	Leclair		MD	Hôpital Fleurimont – Sherbrooke (CHUS)	Sherbrooke, Quebec, Cana	Co-Investigator	
Alexandre	Leclerc			Hôpital Hôtel-Dieu De Lévis	Lévis, Quebec, Canada	Research Assistant	
Hilary	Lee		M.D., FRCPC	Brantford General Hospital	Brantford, Ontario, Canada	Site Co-Investigator	
Rose	Lee		M.Sc PT	St. Michael's Hospital	Toronto, Ontario, Canada	Blinded Outcome Assessor	
Christine	Leger		M.Sc PT	St. Michael's Hospital	Toronto, Ontario, Canada	Interventionist	
Laura	Lehany		M.Physio	Austin Hospital	Victoria, Australia	Blinded Outcome Assessor	
Cassandra	Lemieux		M.Sc PT	Niagara Health St Catherines	St Catherines, Ontario, Canada	Other	
Cindy	Lessard		RN	Hôpital Fleurimont – Sherbrooke (CHUS)	Sherbrooke, Quebec, Cana	Interventionist	
Donna	Leybourne		B.ScN, MBA	Kingston General Hospital	Kingston, Ontario, Canada	Administration	
Sarah	Lohonyai		M.Sc P.T.	Hamilton General Hospital	Hamilton, Ontario, Canada	Interventionist	Blind Outcome Assessor
Elise	Loreto		M.Sc P.T.	Hamilton General Hospital	Hamilton, Ontario, Canada	Interventionist	Blind Outcome Assessor

Supplemental Online Content: Nonauthor Collaborators
*First name, last name, and suffix (if applicable) are required and will appear in PubMed.

*First Name and Middle Initial(s)	*Last Name	*Suffix (eg, Jr, III)	Academic Degrees	Institution	Location (city, state/province, country)	Role or Contribution, eg, chair, principal investigator	Group (if more than 1 Group listed in the byline) and/or Subgroup (eg, Steering Committee)
Ally	Macdonell		B.Physio	Austin Hospital	Victoria, Australia	Research Coordinator	Interventionist
Al	MacVicar		B.Sc PT	Kingston General Hospital	Kingston, Ontario, Canada	Interventionist	
Sue	Mahler		B.Sc PT	Hamilton General Hospital	Hamilton, Ontario, Canada	Blind Outcome Assessor	
Gillian	Manson		B.Sc PT	Juravinski Hospital	Hamilton, Ontario, Canada	Blinded Outcome Assessor	
Joannie	Marchand			Hôpital Fleurimont – Sherbrooke (CHUS)	Sherbrooke, Quebec, Cana	Research Coordinator	
Cara	Mariani		BS	Duke University Hospital	Durham, North Carolina, USA	Research Coordinator	
Emile	Marmen			Hôpital Hôtel-Dieu De Lévis	Lévis, Quebec, Canada	Research Assistant	
Nicole	Marquis			Hôpital Fleurimont – Sherbrooke (CHUS)	Sherbrooke, Quebec, Cana	Interventionist	Other
Albert	Martin		MD	Hôpital du Sacré-Coeur de Montréal	Montréal, Quebec, Canada	Co-Investigator	
Roxanne	Martineau		B.Sc PT	Hôpital du Sacré-Coeur de Montréal	Montréal, Quebec, Canada	Interventionist	
Patricia	Martinez Barrios		M.Sc	Hôpital du Sacré-Coeur de Montréal	Montréal, Quebec, Canada	Research Assistant	
Lyne	Marum		B.Sc PT	Ottawa General Hospital	Ottawa, Ontario, Canada	Interventionist	
David	Maslove		MD, MSc,	Kingston General Hospital	Kingston, Ontario, Canada	Site Co-Investigator	
Matt	McCaffrey		B.Sc PT	Hamilton General Hospital	Hamilton, Ontario, Canada	Blind Outcome Assessor	
Magda	McCaughan		M.Sc PT, MHM	St Joseph's Healthcare Hamilton	Hamilton, Ontario, Canada	Blinded Outcome Assessor	
Ellen	McDonald		B.ScN	Hamilton General Hospital	Hamilton, Ontario, Canada	Research Coordinator	

Supplemental Online Content: Nonauthor Collaborators
*First name, last name, and suffix (if applicable) are required and will appear in PubMed.

***First Name and Middle Initial(s)**	***Last Name**	***Suffix (eg, Jr, III)**	Academic Degrees	Institution	Location (city, state/province, country)	Role or Contribution, eg, chair, principal investigator	Group (if more than 1 Group listed in the byline) and/or Subgroup (eg, Steering Committee)
Luke	McDonald		B.AppSci, M.Physio	Austin Hospital	Victoria, Australia	Interventionist	Blinded Outcome Assessor
Hanna	McHugh		DPT	Duke University Hospital	Durham, North Carolina, USA	Research Assistant	
Fatima	Mehmood		B.Sc	Niagara Health St Catherines	St Catherines, Ontario, Canada	Research Assistant	
Sangeeta	Mehta		M.D. FRCPC	Mount Sinai Hospital	Toronto, Ontario, Canada	Site Principal Investigator	
Masoumeh	Memarzadeh			St Joseph's Healthcare Hamilton	Hamilton, Ontario, Canada	Blinded Outcome Assessor	
Anna	Michalski		MSc PT	St. Michael's Hospital	Toronto, Ontario, Canada	Blinded Outcome Assessor	
Marie	Michelle-Watt		MScAPT	Hôpital Fleurimont – Sherbrooke (CHUS)	Sherbrooke, Quebec, Cana	Administration	
Sydeny	Miezitis		B.Sc	Ottawa Civic Hospital	Ottawa, Ontario, Canada	Research Assistant	
Tina	Millen		RRT	Juravinski Hospital	Hamilton, Ontario, Canada	Research Coordinator	
Angela	Miller		M.Sc P.T.	Hamilton General Hospital	Hamilton, Ontario, Canada	Interventionist	Blind Outcome Assessor
Lexie	Mitris		B.Sci, DPT	Austin Hospital	Victoria, Australia	Blinded Outcome Assessor	
Laura	Montgomery		B.Biomed Sci, DPT	Austin Hospital	Victoria, Australia	Blinded Outcome Assessor	
Kara	Morrison		M.Sc PT	St. Michael's Hospital	Toronto, Ontario, Canada	Interventionist	
Tina	Murphy		M.Sc P.T.	St Joseph's Healthcare Hamilton	Hamilton, Ontario, Canada	Blinded Outcome Assessor	
Coralie	Mus			Hôpital Hôtel-Dieu De Lévis	Lévis, Quebec, Canada	Research Assistant	

*First name, last name, and suffix (if applicable) are required and will appear in PubMed.

*First Name and Middle Initial(s)	*Last Name	*Suffix (eg, Jr, III)	Academic Degrees	Institution	Location (city, state/province, country)	Role or Contribution, eg, chair, principal investigator	Group (if more than 1 Group listed in the byline) and/or Subgroup (eg, Steering Committee)
Zain	Nagaria		MD	University of Maryland	Baltimore, Maryland, USA	Research Assistant	
Celine	Nathoo		M.Sc PT	Mount Sinai Hospital	Toronto, Ontario, Canada	Blinded Outcome Assessor	
Christine	Neilson		B.H.S.c, OTA/PTA	St Joseph's Healthcare Hamilton	Hamilton, Ontario, Canada	Blinded Outcome Assessor	
Kate	Neumann		MPT	London Health Sciences Centre	London, Ontario, Canada	Blinded Outcome Assessor	
Anastasia	Newman		Ph.D PT	Hamilton General Hospital	Hamilton, Ontario, Canada	Interventionist	Blind Outcome Assessor
Randy	Nguyen		M.Sc PT	Hamilton General Hospital	Hamilton, Ontario, Canada	Blind Outcome Assessor	
Diane	Nielsen-Smith		B.A.	Niagara Health St Catherines	St Catherines, Ontario, Canada	Research Assistant	
Alli	Nikolovski		M.Sc PT CRFC	St Joseph's Healthcare Hamilton	Hamilton, Ontario, Canada	Student	
Leigh Ann	Niven		M.Sc PT	Juravinski Hospital	Hamilton, Ontario, Canada	Interventionist	Blind Outcome Assessor
Monia	Noel-Hunter			Hôpital Hôtel-Dieu De Lévis	Lévis, Quebec, Canada	Research Coordinator	
Kristy	Obrovac		M.Sc P.T.	St Joseph's Healthcare Hamilton	Hamilton, Ontario, Canada	Interventionist	Blind Outcome Assessor
Tessa	O'Dea		B.PTB.Exercise and Sport Sci, DPT	Austin Hospital	Victoria, Australia	Blinded Outcome Assessor	
Carina	Orschel		M.Sc PT	St. Michael's Hospital	Toronto, Ontario, Canada	Blinded Outcome Assessor	
Athena	Ovsenek		BA	London Health Sciences Centre	London, Ontario, Canada	Research Assistant	

Supplemental Online Content: Nonauthor Collaborators
*First name, last name, and suffix (if applicable) are required and will appear in PubMed.

***First Name and Middle Initial(s)**	***Last Name**	***Suffix (eg, Jr, III)**	Academic Degrees	Institution	Location (city, state/province, country)	Role or Contribution, eg, chair, principal investigator	Group (if more than 1 Group listed in the byline) and/or Subgroup (eg, Steering Committee)
Kristen	Paguiligan		OTA/PTA	St. Michael's Hospital	Toronto, Ontario, Canada	Interventionist	
Rebecca	Parsons		B.Sci, DPT	Austin Hospital	Victoria, Australia	Blinded Outcome Assessor	
Harsh	Patel		MD	University of Maryland	Baltimore, Maryland, USA	Research Assistant	
Sal	Patten		B.Sc PT	Ottawa Civic Hospital	Ottawa, Ontario, Canada	Interventionist	Blinded Outcome Assessor
Lisa	Patterson			Niagara Health St Catherines	St Catherines, Ontario, Canada	Research Coordinator	
Stephanie	Pedley		OTA/PTA	Brantford General Hospital	Brantford, Ontario, Canada	Blinded Outcome Assessor	
Dominique	Pellerin		MScAPT	Hôpital Fleurimont – Sherbrooke (CHUS)	Sherbrooke, Quebec, Cana	Research Coordinator	
Joseph	Pellizzari		Ph.D C.Psych	St Joseph's Healthcare Hamilton	Hamilton, Ontario, Canada	Clinical Psychologist	
Alexandre	Pepin			Hôpital Hôtel-Dieu De Lévis	Lévis, Quebec, Canada	Research Assistant	
Kathleen	Perri		M.Sc PT	Kingston General Hospital	Kingston, Ontario, Canada	Interventionist	
Christy	Peterson		BS	Duke University Hospital	Durham, North Carolina, USA	Research Coordinator	
Jessica	Pilon-Bignell		M.Sc PT	Hamilton General Hospital	Hamilton, Ontario, Canada	Blind Outcome Assessor	
Celeste	Plant		B,App Sci, M.Physio	Austin Hospital	Victoria, Australia	Blinded Outcome Assessor	Interventionist
Andreea	Podgoreanu		BS	Duke University Hospital	Durham, North Carolina, USA	Research Coordinator	
Makena	Pook		B.HSc	Hamilton General Hospital	Hamilton, Ontario, Canada	Research Coordinator	

Supplemental Online Content: Nonauthor Collaborators
*First name, last name, and suffix (if applicable) are required and will appear in PubMed.

***First Name and Middle Initial(s)**	***Last Name**	***Suffix (eg, Jr, III)**	Academic Degrees	Institution	Location (city, state/province, country)	Role or Contribution, eg, chair, principal investigator	Group (if more than 1 Group listed in the byline) and/or Subgroup (eg, Steering Committee)
Rebecca	Porteous		B.N.Sc	Ottawa Civic Hospital	Ottawa, Ontario, Canada	Research Coordinator	
Tara	Porter		OTA/PTA	Brantford General Hospital	Brantford, Ontario, Canada	Interventionist	
Yannick	Poulin		MD	Hôpital Fleurimont – Sherbrooke (CHUS)	Sherbrooke, Quebec, Cana	Co-Investigator	
Stacey	Priest		M.Sc	St Joseph's Healthcare Hamilton	Hamilton, Ontario, Canada	Methods Centre	
Miranda	Prince			St Joseph's Healthcare Hamilton	Hamilton, Ontario, Canada	Blinded Outcome Assessor	
Phillip	Quessy		MScAPT	Hôpital Hôtel-Dieu De Lévis	Lévis, Quebec, Canada	Interventionist	Blinded Outcome Assessor
Chris	Ramlu		B.Sci, B.Physio, M.Physio	Austin Hospital	Victoria, Australia	Blinded Outcome Assessor	
Liv	Ramsden		DPT	Austin Hospital	Victoria, Australia	Interventionist	
Kathleen	Reardon		B.Sc PT	Niagara Health St Catherines	St Catherines, Ontario, Canada	Interventionist	
Valerie	Reid		B.Sc PT	Ottawa General Hospital	Ottawa, Ontario, Canada	Blinded Outcome Assessor	
Sarah	Retica		B.Sci, DPT	Austin Hospital	Victoria, Australia	Research Coordinator	Interventionist
Tracey	Reynolds			Hamilton General Hospital	Hamilton, Ontario, Canada	Administration	
Melissa	Richardson		M.Sc P.T.	Hamilton General Hospital	Hamilton, Ontario, Canada	Interventionist	Blind Outcome Assessor
Hayden	Rigsbee		BA	Duke University Hospital	Durham, North Carolina, USA	Research Coordinator	
Caroline	Rivard		B.Sc PT	Ottawa General Hospital	Ottawa, Ontario, Canada	Blinded Outcome Assessor	

Supplemental Online Content: Nonauthor Collaborators
*First name, last name, and suffix (if applicable) are required and will appear in PubMed.

***First Name and Middle Initial(s)**	***Last Name**	***Suffix (eg, Jr, III)**	Academic Degrees	Institution	Location (city, state/province, country)	Role or Contribution, eg, chair, principal investigator	Group (if more than 1 Group listed in the byline) and/or Subgroup (eg, Steering Committee)
Courtney	Robb		B.H.Sc	Brantford General Hospital	Brantford, Ontario, Canada	Research Assistant	
Amber	Robertson		M.Sc PT	St. Michael's Hospital	Toronto, Ontario, Canada	Interventionist	
Isabelle	Robitaille		MScAPT	Hôpital Hôtel-Dieu De Lévis	Lévis, Quebec, Canada	Interventionist	
Mylene	Rochon		MScAPT	Hôpital Hôtel-Dieu De Lévis	Lévis, Quebec, Canada	Interventionist	
Valeriya	Rodionova		MD	University of Maryland	Baltimore, Maryland, USA	Research Coordinator	
Thomas	Rollinson		B.Physio	Austin Hospital	Victoria, Australia	Interventionist	Research Coordinator
Angela	Romero		PCA	Kingston General Hospital	Kingston, Ontario, Canada	Interventionist	
Joleen	Rose		B.Sci, B.Physio	Austin Hospital	Victoria, Australia	Interventionist	
Robin	Roy			Hôpital Hôtel-Dieu De Lévis	Lévis, Quebec, Canada	ICU Nurse	
Anna	Rozenberg		M.D.	Brantford General Hospital	Brantford, Ontario, Canada	Site Co-Investigator	
Justine	Ruel			Hôpital Hôtel-Dieu De Lévis	Lévis, Quebec, Canada	Research Assistant	
Karen	Saab		B.Sc PT	Ottawa General Hospital	Ottawa, Ontario, Canada	Blinded Outcome Assessor	
Laurie	Sadowski		M.Sc	Niagara Health St Catherines	St Catherines, Ontario, Canada	Research Assistant	
Uzma	Saeed		CRA, M.B	Hamilton General Hospital	Hamilton, Ontario, Canada	Research Coordinator	
Eric	Samson		MScAPT	Hôpital Hôtel-Dieu De Lévis	Lévis, Quebec, Canada	Head of PT Service	
Gyan	Sandhu		BScN	St. Michael's Hospital	Toronto, Ontario, Canada	Research Coordinator	
Marlene	Santos		M.Sc	St. Michael's Hospital	Toronto, Ontario, Canada	Research Coordinator	
Kristina	Saric		M.Sc PT	Hamilton General Hospital	Hamilton, Ontario, Canada	Blind Outcome Assessor	

Supplemental Online Content: Nonauthor Collaborators
*First name, last name, and suffix (if applicable) are required and will appear in PubMed.

***First Name and Middle Initial(s)**	***Last Name**	***Suffix (eg, Jr, III)**	Academic Degrees	Institution	Location (city, state/province, country)	Role or Contribution, eg, chair, principal investigator	Group (if more than 1 Group listed in the byline) and/or Subgroup (eg, Steering Committee)
Ashley	Sawyer		B.H.Sc	St Joseph's Healthcare Hamilton	Hamilton, Ontario, Canada	Methods Centre	
Alicja	Schultz		BS, MBA	Duke University Hospital	Durham, North Carolina, USA	Research Coordinator	
Kanaan	Shaath			Hôpital Hôtel-Dieu De Lévis	Lévis, Quebec, Canada	Research Assistant	
Sumesh	Shah		CCRP	Mount Sinai Hospital	Toronto, Ontario, Canada	Research Coordinator	
Stephanie	Sibley		MSc, MD, I	Kingston General Hospital	Kingston, Ontario, Canada	Site Co-Investigator	
Christine	Smit			London Health Sciences Centre	London, Ontario, Canada	Blinded Outcome Assessor	
Kimberley	Smith		B.Sc OT	Kingston General Hospital	Kingston, Ontario, Canada	Administration	
Orla	Smith		PhD	St. Michael's Hospital	Toronto, Ontario, Canada	Research Coordinator	
Pascal	Smith			Hôpital Hôtel-Dieu De Lévis	Lévis, Quebec, Canada	Research Coordinator	
Olivia	So		M.Sc PT	St. Michael's Hospital	Toronto, Ontario, Canada	Interventionist	
Jessica	Sorensen		M.Sc PT	Juravinski Hospital	Hamilton, Ontario, Canada	Blinded Outcome Assessor	Interventionist
Krysten	Spurrier		OTR/L	Duke University Hospital	Durham, North Carolina, USA	Interventionist	
Kristy	Steeves		M.Sc PT	Ottawa General Hospital	Ottawa, Ontario, Canada	Interventionist	Blinded Outcome Assessor
Nicholle	Stein		DPT	Duke University Hospital	Durham, North Carolina, USA	Research Assistant	
Gabriel	Streisfeld		DPT	Duke University Hospital	Durham, North Carolina, USA	Research Assistant	Interventionist
Susan	Surca		M.Sc PT	Kingston General Hospital	Kingston, Ontario, Canada	Blinded Outcome Assessor	

Supplemental Online Content: Nonauthor Collaborators
*First name, last name, and suffix (if applicable) are required and will appear in PubMed.

***First Name and Middle Initial(s)**	***Last Name**	***Suffix (eg, Jr, III)**	Academic Degrees	Institution	Location (city, state/province, country)	Role or Contribution, eg, chair, principal investigator	Group (if more than 1 Group listed in the byline) and/or Subgroup (eg, Steering Committee)
Parth	Suthar		M.Sc PT	St Joseph's Healthcare Hamilton	Hamilton, Ontario, Canada	Blinded Outcome Assessor	
Katherine	Sweeney		RN, MSN	Duke University Hospital	Durham, North Carolina, USA	Research Coordinator	
Marilyn	Swinton		M.Sc	St Joseph's Healthcare Hamilton	Hamilton, Ontario, Canada	Methods Centre	
Audrey	Tanguay		B.Erg	Hôpital Hôtel-Dieu De Lévis	Lévis, Quebec, Canada	PT Coordinator	
Victoria	Thibault			Hôpital Hôtel-Dieu De Lévis	Lévis, Quebec, Canada	Research Assistant	
Francisco	Torres		MD	University of Maryland	Baltimore, Maryland, USA	Research Coordinator	
Bao Tran	Tran			Hôpital Hôtel-Dieu De Lévis	Lévis, Quebec, Canada	Research Assistant	
Deana	Tran		MD	University of Maryland	Baltimore, Maryland, USA	Research Assistant	
Lauren	VanDerLelie		M.Sc PT	Hamilton General Hospital	Hamilton, Ontario, Canada	Blind Outcome Assessor	
Lori	Vantfoort			London Health Sciences Centre	London, Ontario, Canada	Blinded Outcome Assessor	
Anu	Varghese		MD	University of Maryland	Baltimore, Maryland, USA	Research Coordinator	
Hannah	Verspey		B.Sci, B.Physio	Austin Hospital	Victoria, Australia	Interventionist	
Josh	Vogel		DPT	University of Maryland	Baltimore, Maryland, USA	Interventionist	
Jennifer	Wagar		B.Sc PT	Niagara Health St Catherines	St Catherines, Ontario, Canada	Blinded Outcome Assessor	
Nicke	Warzee		B.Sc PT	Ottawa General Hospital	Ottawa, Ontario, Canada	Blinded Outcome Assessor	
Irene	Watpool		BScN, RN	Ottawa General Hospital	Ottawa, Ontario, Canada	Research Coordinator	
Chris	Wells		PhD, PT, CCS, ATC	University of Maryland	Baltimore, Maryland, USA	Interventionist	

Supplemental Online Content: Nonauthor Collaborators
*First name, last name, and suffix (if applicable) are required and will appear in PubMed.

***First Name and Middle Initial(s)**	***Last Name**	***Suffix (eg, Jr, III)**	Academic Degrees	Institution	Location (city, state/province, country)	Role or Contribution, eg, chair, principal investigator	Group (if more than 1 Group listed in the byline) and/or Subgroup (eg, Steering Committee)
Alyssa	Whelan		M.Sc PT	Kingston General Hospital	Kingston, Ontario, Canada	Interventionist	
Lane	White		DPT	Duke University Hospital	Durham, North Carolina, USA	Interventionist	
Miranda	Wiens		M.Sc P.T.	Hamilton General Hospital	Hamilton, Ontario, Canada	Interventionist	Blind Outcome Assessor
Virginie	Williams		PhD	Hôpital du Sacré-Coeur de Montréal	Montréal, Quebec, Canada	Research Coordinator	
David	Williamson		PhD	Hôpital du Sacré-Coeur de Montréal	Montréal, Quebec, Canada	Co-Investigator	
Louise M	Williamson		MOT	Duke University Hospital	Durham, North Carolina, USA	Interventionist	Blinded Outcome Assessor
Laura	Winters		B.Sc	London Health Sciences Centre	London, Ontario, Canada	Research Assistant	
Paul	Wischmeyer		MD	Duke University Hospital	Durham, North Carolina, USA	Site Co-Investigator	
Ashley	Woodroffe-McClintock		M.Sc PT	Kingston General Hospital	Kingston, Ontario, Canada	Blinded Outcome Assessor	
Tony	Yuan		B.Comm, B.Eng, M.Sc PT	Austin Hospital	Victoria, Australia	Interventionist	Blinded Outcome Assessor
Meaghan	Zsolt		M.Sc PT	St. Michael's Hospital	Toronto, Ontario, Canada	Blinded Outcome Assessor	
