# Supplementary material for: Cost-Effectiveness of In-Bed Cycling and Routine Physiotherapy for Patients Receiving Mechanical Ventilation
Source: JAMA Netw Open. 2025 Sep 8;8(9):e2529399. doi: 10.1001/jamanetworkopen.2025.29399 (PMC12418132; doi:10.1001/jamanetworkopen.2025.29399)
Supplement: Supplement 3. — Data Sharing Statement [file jamanetwopen-e2529399-s003.pdf]

Data Sharing Statement

Tarride. Cost-Effectiveness of In-Bed Cycling and Routine Physiotherapy for Patients Receiving Mechanical Ventilation. *JAMA Netw Open*. Published September 08, 2025. doi:10.1001/jamanetworkopen.2025.29399

Data
Data available: Yes
Data types: Deidentified participant data, Data dictionary, Other (please specify)
Additional Information: Partial data sets
How to access data: Data requests through the CYCLE steering committee chair, Dr. Michelle Kho, khome@mcmaster.ca. Data will be provided according to prespecified hypotheses including only data relevant to the research question. The data will be available following the submission of a research question, approval by the CYCLE steering committee, and execution of a data-sharing agreement. Salary cost-recovery for the analyst's time to develop the dataset may be required.
When available: beginning date: 07-01-2026, end date: 07-01-2029

Supporting Documents
Document types: None

Additional Information
Who can access the data: The data will be available following approval by the CYCLE steering committee, an appropriate research hypothesis developed, and execution of a data-sharing agreement. Salary cost-recovery for the analyst's time to develop the dataset may be required.
Types of analyses: The data can only be used for the purpose pre-specified in the application submitted to the CYCLE steering committee.
Mechanisms of data availability: The data will be available following the submission of a research question, approval by the CYCLE steering committee, and execution of a data-sharing agreement. Salary cost-recovery for the analyst's time to develop the dataset may be required.
